# Design and Validation of Exoskeleton Actuated by Soft Modules toward Neurorehabilitation—Vision-Based Control for Precise Reaching Motion of Upper Limb

**DOI:** 10.3389/fnins.2017.00352

**Published:** 2017-07-07

**Authors:** Victoria W. Oguntosin, Yoshiki Mori, Hyejong Kim, Slawomir J. Nasuto, Sadao Kawamura, Yoshikatsu Hayashi

**Affiliations:** ^1^Brain Embodiment Lab, Biomedical Engineering, School of Biological Sciences, University of ReadingReading, United Kingdom; ^2^Department of Robotics, Ritsumeikan UniversityShiga, Japan

**Keywords:** 3D printed exoskeleton, soft actuators, compliant assistance, active and passive mechanisms, gravity compensation, reaching motion

## Abstract

We demonstrated the design, production, and functional properties of the Exoskeleton Actuated by the Soft Modules (EAsoftM). Integrating the 3D printed exoskeleton with passive joints to compensate gravity and with active joints to rotate the shoulder and elbow joints resulted in ultra-light system that could assist planar reaching motion by using the vision-based control law. The EAsoftM can support the reaching motion with compliance realized by the soft materials and pneumatic actuation. In addition, the vision-based control law has been proposed for the precise control over the target reaching motion within the millimeter scale. Aiming at rehabilitation exercise for individuals, typically soft actuators have been developed for relatively small motions, such as grasping motion, and one of the challenges has been to extend their use for a wider range reaching motion. The proposed EAsoftM presented one possible solution for this challenge by transmitting the torque effectively along the anatomically aligned with a human body exoskeleton. The proposed integrated systems will be an ideal solution for neurorehabilitation where affordable, wearable, and portable systems are required to be customized for individuals with specific motor impairments.

## 1. Introduction

Although an increased effort has been placed on the recovery process of patients following a stroke, with recent advances in technology for monitoring the brain functions, lack of human resources in therapeutic training of patients implies that patients generally may not reach their full recovery potential when discharged from hospital following initial rehabilitation (Loureiro and Harwin, 2007). Neurological illness often manifests in clinical condition resulting in weakness of the muscles controlling the elbow and rehabilitation devices are used in order to help in recovery of muscular power (Maciejasz et al., 2014). In stroke patients, damage to the motor-, and somato-sensory cortices, makes it difficult to control and move the arm, forearm, and fingers. These patients often use assistive devices and therapy to encourage them to use their damaged limb so that the re-growth of a neural circuitry can take place that would eventually aid recovery through neuro-plasticity (Jack et al., 2001). Therefore, there is a growing interest in using robotic devices to deliver effective assistance. The aim of the robotic assistance is to provide a set of intensive and repetitive therapies to enhance the motor recovery of patients, decreasing the amount of work of a therapist. It has been found that it is important for a patient to undergo continuous exercise for successful rehabilitation (Heo et al., 2012) which robots are able to provide.

Typically, rehabilitation methods are either highly human intensive or involve attachment of rigid robotic system controlling the upper limb. Robot-based rehabilitation has been shown to have positive effects by reducing impairment (Loureiro et al., 2014). Robots provide an acceptable performance measurement test and control of the amount of exercise delivered to the subject (Kutner et al., 2014). Hard robotic structures are often based on joints connected by rigid links; this makes them heavy, with expensive and complicated control. In addition, these robotic systems are less compliant than the joints they actuate making it difficult to be directly attached to the human body unless through the use of specialized end effectors (Krebs et al., 2000), thereby mitigating their use for personal use by patients. To overcome these challenges, safe human robotic interactions, and inexpensive design can be achieved through a soft wearable robotic device (Tondu and Lopez, 2000; Tsagarakis and Caldwell, 2000).

A novel approach to rehabilitation is making use of inexpensive and soft actuators. Soft actuators are usually actuated with compressed air or SMAs (Shape Memory Alloy) (Chiu et al., 2013). SMA actuation is accomplished by embedding the trained alloy into the silicone rubber and using electric current to produce heat to deform the robot to the trained shape. Soft robots can be realized with the use of McKibben artificial muscles to produce a compliant motion similar to the skeletal muscle (Tsagarakis and Caldwell, 2000) and are actuated using a pneumatic source (Takagi et al., 2009).

Another novel approach uses embedded pneumatic networks of channels in elastomers (Ilievski et al., 2011) to achieve bending and crawling motions (Ilievski et al., 2011). Soft rehabilitation robots have been developed to generate assistive force for grasping (Shepherd et al., 2011) and gait rehabilitation (Shepherd et al., 2011). They make use of air channels embedded in elastomers to perform bending motions similar to the motion of the fingers. Other robots composed of silicone rubber for angular displacement (Sun et al., 2013) and as a 2D (Marchese et al., 2014) or 3D (Martinez et al., 2013) manipulators have been developed. An upper limb orthosis called Orthojacket actuated by pneumatics and designed for elbow rehabilitation has been developed (Schulz et al., 2011).

There have been a lot of research in developing and implementation of robotic devices for upper limb rehabilitation especially index and finger rehabilitation in the form of exoskeletons (Chiri et al., 2012; Maciejasz et al., 2014). These hand exoskeletons make use of cables (Dovat et al., 2008) and rubber (Polygerinos et al., 2015) for actuation purposes. Virtual reality has been used together with these hand exoskeletons to further aid in recovery (Ueki et al., 2012). Target tracking have also been incorporated in order to increase motivation and co-operation from the user as well as individualize exercise difficulty depending on strength the user (Jack et al., 2001).

In traditional, robotic rehabilitation systems interacting with the patient using a single distal attachment point on the forearm by means of an orthosis [for example, MIT-MANUS (Krebs et al., 2004)], exercises are defined in the XYZ Cartesian space relative to the robots single end-effector attachment point, and the assistance magnitude is modulated using impedance/admittance control schemes in the robot task-space. To support comfortably upper limb flexion and extension, WREX, Wilmington Robotic EXoskeleton, was designed at A. I. DuPont Hospital for Children to provide assistance for the wide range of natural motion in daily settings (Sanchez et al., 2006; Tariq Rahman et al., 2006). The JAECO WREX is a light weight exoskeleton with two links and four degrees of motion that approximates normal human anatomy. It incorporates elastic band elevation assists for both the shoulder and elbow to eliminate influence of gravity on the extremity (Sanchez et al., 2006; Tariq Rahman et al., 2006). The unique design of the shoulder and elbow joints allows for a significant improvement in the available range of motion when compared to other assistive devices.

A new type of wearable robots called soft exoskeletons or exosuits is becoming more popular, aiming at therapeutic intervention of upper limb by mirroring the motion of the other limb (Panasonic, 2005). Removing the external metal frame, soft exoskeletons become compliant, much lighter, and could be significantly less expensive than rigid frame wearable robots. However, soft exoskeleton's main drawback is the same as their main strength: they have no external rigid frame to support the body parts and transfer force effectively from actuator to some area of the body. We think that the effective transfer of the force should take into account the anatomical structure of limbs and joints, requiring the solid structure of the assistive device in order not to add unnecessary strain on the body parts that might damage patient joints, muscles, or tendons.

As a unique approach to a lightweight and flexible robotic structure, inflatable structures have been developed for the robotic arm as an attractive alternative method of imbuing robots with the desired lightness. Kim et al. developed the robotic arm that comprised of the inflatable links, air bag actuators, and ABS joint structures (Kim et al., 2015). Also, the novel control schemes have been applied on the basis of visual feedback control, which do not require the precise inverse kinematics for the position control (Nishida and Kawamura, 2012).

In our study, aiming at the reaching motion of the upper limb, we developed and validated the Exoskeleton Actuated by Soft Modules (EASoftM) to fulfill the four conditions; (1) A few degrees of freedom, (2) Precise motion control, (3) Wearable assistive robots, (4) Compliant assistance. We proposed the exoskeleton aligned with the anatomical structure, actuated by the soft modules located at the joint position, and the soft actuators realize the compliant motion based on pneumatic actuation and visco-elastic properties of soft materials such as plastic and rubber.

From the patient perspective, the ultra-light EASoftM offers upper limb assistance, i.e., it can assist the reaching motion of the upper limb, supporting the movement of the elbow and shoulder, and can record trajectories of hand reaching a target based on the visual feedback control. It also has high compliance and high safety integrity from the perspective of the patient, because it is entirely composed of rubber and soft modules, and attached to the body via Velcro straps. We first describe the design specification of the EASoftM with the scene setting (Sections 2.1 and 2.2), and secondly describe the detailed kinematics and the vision-based control schemes (Sections 3.1–3.3). After explaining the production of the plastic modules (Section 3.4), their torque characteristics and reliability were presented (Section 4). In the Result section (Section 5.1), a series of experiments is described for the motion of one and two degrees of freedom, first tested by using the weight. Finally, we presented the participant experiments, demonstrating the closed-loop reaching motion on precise position control (Section 5.2). The General discussion is given in Section 6.

As a contribution to neurorehabilitation, the EASoftM can be integrated with the Brain Computer Interface, for example, for detecting the motor command generation by online electroencephalogram (EEG) analysis, in order for the EASoftM to guide the hand to a target position in a rehabilitation therapy. The advantage of using EASoftM is that it does not include any motors which generate the elecro-magnetic waves which inevitably influence the electric signals from brain activity.

## 2. Design and specifications

### 2.1. Scene setting for rehabilitation

To minimize detrimental effects of technology on the rehabilitation process, some of the technical requirements present in the design of most rehabilitation robots take into consideration the ergonomics of the system and its ability to cope with a variety of patient demographic and anthropomorphic parameters. An imperative requirement in the design of the human machine interface is that it should mimic behavior of the human therapist. That is, it should be compliant when assisting movement, provide full support within the patients passive range of motion and nourish the patient's confidence and motivation levels through goal-oriented motion with informative biofeedback.

Reaching motion is one of the most critical behaviors in our daily life at home; at the table, we reach and grasp a cup to drink, and in the kitchen we reach for the spice jars or pots. Thus, it is important to assist the motion for reaching purposes. required system characteristics can be decomposed into the following;

1. Attachable to chairs: A whole unit should be easy to be attached to a chair by the mount base.
2. Gravity compensation: An equilibrium point of the upper limb should be shifted to match the desired work space for reaching motion (Sanchez et al., 2006; Tariq Rahman et al., 2006).
3. Identification of the target: A target position should be programmed to be fed into the control law.
4. Guidance of the wrist toward the target: Active joints should provide appropriate torque to rotate the shoulder and elbow to assist the reaching motion.
5. Allowance for small deviation from the planned trajectories: Passive joints should should accommodate small spontaneous motion with compliance, and soft modules should provide compliance naturally by contained air and soft material.

We aim at rehabilitation for people with neuromuscular weakness such as muscle disease, cerebral palsy, spinal cord injury, and stroke, that effect the spontaneous motion of upper limbs. Also, given a proper balance between passive and active joints, the proposed system should provide an appropriate active assistance in guiding the hand to a target position. The integrated system should act as a functional aid in activities of daily living ideally at home, serving as a cost effective exercise/therapy device for people recovering from stroke.

### 2.2. Design specifications

All of the considerations in Section 2.1 lead to five main specifications to be fulfilled by the Exoskeleton Actuated by Soft Modules which can assist the reaching motion;

1. Gravity compensation using exoskeleton: For any actuator, it is challenging to lift up the upper limb against gravity. Thus, the passive joints should be applied to shift the equilibrium position of upper limb (Sanchez et al., 2006; Tariq Rahman et al., 2006).
2. Degrees of freedom: Two passive joints should shift the equilibrium plane of the upper limb, and two active joints should guide the wrist to the target position.
3. Viscoelastic property for passive motion: The assistance by the exoskeleton should be controlled compliantly by viscoelastic properties of air and materials used in the actuators.
4. Structural transparency for active motion: Structural transparency should be such that the mass, viscosity and elasticity of the robotic arm are so small that patients performing spontaneous movement does not feel resistance in interactions with the robotic arm.
5. Vision-based control law: Control law should not require the encoders to decrease the total weight, and to increase the precision in reaching a target, the control law should not rely on the precise inverse kinematics of the exoskeleton.

## 3. Design, material, and methods

Fulfilling the design specifications in the Section 2.2 in order to assist comfortable flexion and extension of the upper limb, we mainly concentrated on three characteristics; (1) A few degrees of freedom; anatomically structured exoskeleton with the parallel links for gravity compensation, (2) Compliant assistance: soft modules to actuate the elbow and shoulder joints, and (3) Precise motion control: visual control loop to guide the hand to the target at high precision.

### 3.1. Kinematic structure of exoskeleton actuated by soft modules (EASoftM)

**Figure 1:**
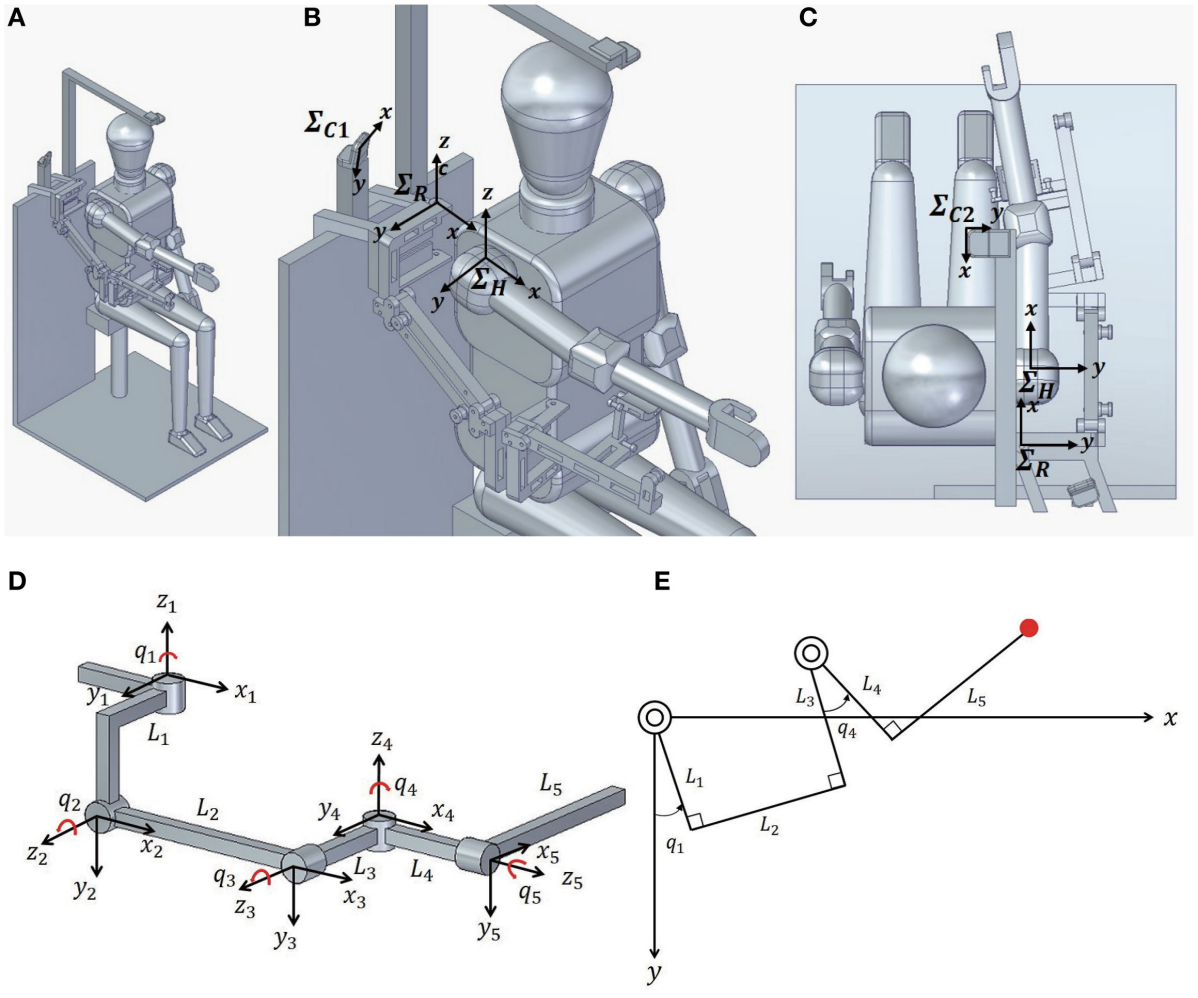
Kinematic structure of Exoskeleton Actuated by Soft Modules (EASoftM) **(A)** Overall structure of the proposed EASoftM with a participant. **(B)** Close-up picture of upper body. $\underset{R}{\sum }$ represents the base coordinate of the robot, $\underset{C1}{\sum }$ the camera coordinate used in the vision-based control law, and $\underset{C2}{\sum }$ the camera coordinate to record the trajectories of the end-effector. The exoskeleton has two parallel links and four degrees of motion that approximates normal human anatomy of upper limb. **(C)** Overall structure of the proposed EASoftM (view from top). The attachment to the upper limb was set up at the end of exoskeleton to support the wrist of a participant. **(D)** Denavit-Hartenberg parameters of exoskeleton structure. The soft modules are attached to the joints (*q*_1_ and *q*_4_) to rotate the joints, and the rubber bands are attached to the parallel structure of link *L*_2_ and *L*_5_ to fix the joint angles of *q*_2_ and *q*_5_ within a certain range, resulting in compensation of the gravity. **(E)** Kinematic structure of exoskeleton in 2D work plane. As the passive joints (*q*_2_ and *q*_5_) lift up the upper limb,the active joints rotates shoulder and elbow (*q*_1_ and *q*_4_) to make a reaching motion possible within this 2D plane.

The overall structure of the proposed EASoftM is given in Figure 1 with a schematic representation of a participant, where $\underset{R}{\sum }$ represents the base coordinate of the robot, $\underset{C1}{\sum }$ the camera coordinate used in the vision-based control law, and $\underset{C2}{\sum }$ the camera coordinate to record the trajectories of the end-effector. The exoskeleton has two parallel links and four degrees of motion that approximate normal human anatomy of the upper limb. The attachment to the upper limb was set up at Robot link *L*_5_ to support the wrist of a participant.

The kinematic structure exoskeleton is given in Figure 1D with DH (Denavit-Hartenberg) parameters in Table 1 (Denavit and Hartenberg, 1955). The length of the links is provided in Table 2. Four joints were represented by *q* where *q*_1_ and *q*_4_ were active joints and *q*_2_ = −*q*_3_ (parallel link) and *q*_5_ were passive joints.

**Table 1:** DH parameters for the kinematic structure.

***i***	***a*_*i*−1_**	**α_*i*−1_**	***d*_*i*_**	**θ_*i*_**
1	0	0	0	*q*_1_
2	0	$-\frac{\pi }{2}$	*L*_1_	*q*_2_
3	*L*_2_	0	0	*q*_3_
4	0	$\frac{\pi }{2}$	−*L*_3_	*q*_4_
5	0	$-\frac{\pi }{2}$	*L*_4_	*q*_5_
E	*L*_5_	0	0	0

**Table 2:** Length of links of exoskeleton.

***L*_1_ [mm]**	***L*_2_ [mm]**	***L*_3_ [mm]**	***L*_4_ [mm]**	***L*_5_ [mm]**
100	210	100	100	210

Before actuation of the soft modules, these two passive joints, *q*_2_ and *q*_5_ were adjusted by the elastic property of the rubber band in order to shift the equilibrium point of upper limb by compensating the gravity. Two soft modules were attached to *q*_1_ and *q*_4_ of the exoskeleton to actuate pneumatically the rotation of joints.

By combining the passive and active joints, we can predetermine the 2D work plane, in which two active joints guide the wrist to the target position. The soft actuators made of polyethylene were attached to individual joints of exoskeleton, providing the visco-elastic property in guidance of motion. All the parts were printed by the 3D printer using ABS plastic. The ultra-light polyethylene modules were used as ideal material for designable and wearable soft robotic modules.

### 3.2. Control schemes of exoskeleton actuated by soft modules (EASoftM)

**Figure 2:**
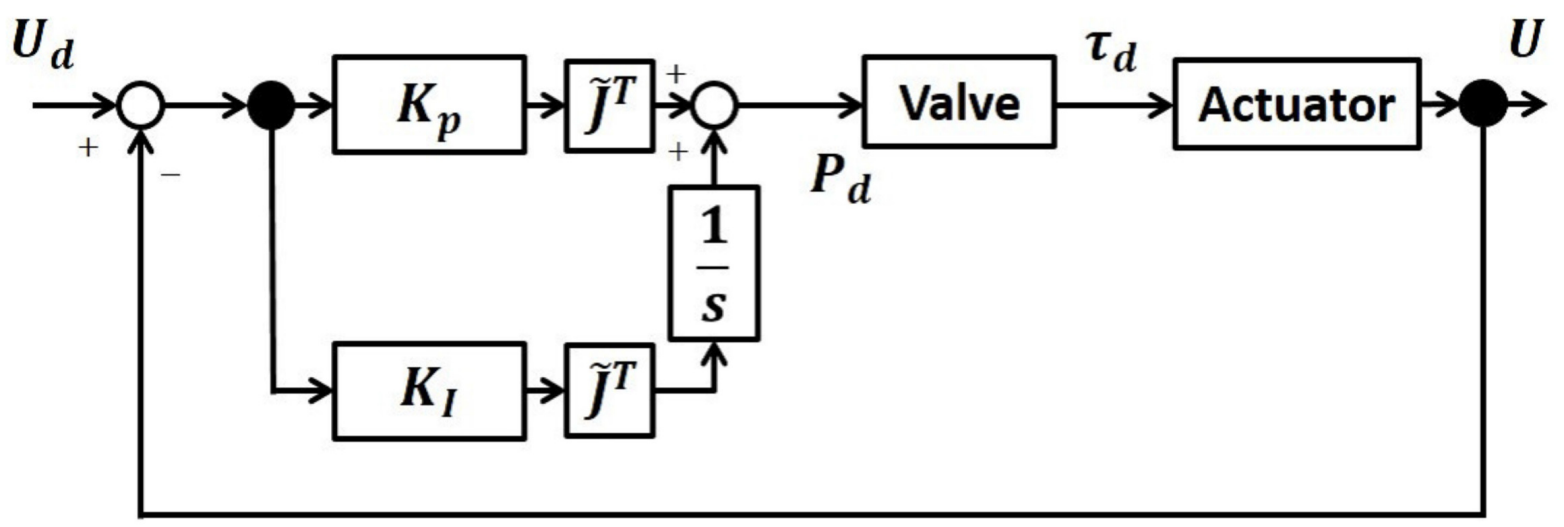
Block Diagram of the control law. Based on the direct transformations from visual space to actuator space, the proposed control scheme will allow a robot to execute visually directed reaching motion with its end-effector without reference to its precise kinematics and necessity to calculate the inverse kinematics.

This study used a control scheme which exploited direct visual-motor loops in the feedback control to actuate robotic arms (Nishida and Kawamura, 2012). Based on the transformations from visual space to actuator space, the proposed control scheme will allow a robot to execute visually directed reaching motion with its end-effector without reference to its precise kinematics and necessity to calculate the inverse kinematics. The block diagram of the control law is given in Figure 2.

Specifically, we apply the Proportional and Integral (PI) feedback control laws to the relative distance between the target and the end effector of the exoskeleton in the 2D work plane defined in Figure 1E. First, we calculate the Jacobian of the exoskeleton in this 2D plane (Figure 1E), which is used in the control law. In this 2D work plane, the position of the end-effector is given by

(1) $$\begin{array}{l} \text{X}=\left[\begin{array}{c} x\\ y \end{array}\right]\\ =\left[\begin{array}{c} \left(L_{1}-L_{3}\right)\text{sin}q_{1}+L_{2}\text{cos}q_{1}+L_{4}\text{sin}\left(q_{1}+q_{4}\right)\\ +\;L_{5}\text{cos}\left(q_{1}+q_{4}\right)\\ \left(L_{1}-L_{3}\right)\text{cos}q_{1}-L_{2}\text{sin}q_{1}+L_{4}\text{cos}\left(q_{1}+q_{4}\right)\\ +\;L_{5}\text{sin}\left(q_{1}+q_{4}\right) \end{array}\right] \end{array}$$

Thus, the Jacobian, **J**_(**q**)_ is given in the 2D plane by **Ẋ** = **J**_(**q**)_$\dot{\text{q}}$, where $\dot{\text{q}}={\left[{\dot{\text{q}}}_{1}\;{\dot{\text{q}}}_{4}\right]}^{\text{T}}$. **J**_(**q**)_ is given by

(2) $$\begin{array}{l} {\text{J}}_{\left(\text{q}\right)}=\left[\begin{array}{cc} a_{11} & a_{12}\\ a_{21} & a_{22} \end{array}\right], \end{array}$$

$$\begin{array}{l} a_{11}=\left(L_{1}-L_{3}\right)\text{cos}q_{1}-L_{2}\text{sin}q_{1}+L_{4}\text{cos}\left(q_{1}+q_{4}\right)\\ -\;L_{5}\text{sin}\left(q_{1}+q_{4}\right)\\ a_{12}=L_{4}\text{cos}\left(q_{1}+q_{4}\right)-L_{5}\text{sin}\left(q_{1}+q_{4}\right)\\ a_{21}=-\left(L_{1}-L_{3}\right)\text{sin}q_{1}-L_{2}\text{cos}q_{1}-L_{4}\text{sin}\left(q_{1}+q_{4}\right)\\ +\;L_{5}\text{cos}\left(q_{1}+q_{4}\right)\\ a_{12}=-L_{4}\text{sin}\left(q_{1}+q_{4}\right)+L_{5}\text{cos}\left(q_{1}+q_{4}\right) \end{array}$$

Generally speaking, a **PI** control law for the target torque is given by

(3) $$\begin{array}{l} \tau_{\text{d}}\left(\text{t}\right)={\text{K}}_{\text{P}}{\text{J}}_{\left(\text{q}\right)}^{\text{T}}\Delta \text{X}+\int_{0}^{\text{t}}{\text{K}}_{\text{I}}{\text{J}}_{\left(\text{q}\right)}^{\text{T}}\Delta \text{X}\text{d}\delta , \end{array}$$

with τ_**d**_(**t**): Target torque, ${\text{J}}_{\left(\text{q}\right)}^{\text{T}}$: Transposed Jacobian, **K**_**P**_: Partial gain parameter, Δ**X**: Relative distance between the target and the end-effector in the robot coordinate, Σ_*R*_, **K**_**I**_: Integral gain parameter.

Here, we need to perform the coordinate transformation from the camera coordinate, Σ_*C*1_, to the robotic coordinate, Σ_*R*_ (Figure 1). Thus, Δ***X*** is calculated in the following way;

(4) $$\begin{array}{l} \Delta \text{X}={\text{C}}_{1}{\text{W}}_{1}\Delta {\text{U}}_{1}, \end{array}$$

where **C_1_** is the coordinate direction transformation matrix, **W_1_** is the coordinate rotation transformation matrix, and the relative distance between the target and the end-effector, Δ**U_1_** is represented by

(5) $$\begin{array}{l} \Delta {\text{U}}_{1}=\left[\begin{array}{c} u_{d1}-u_{1}\\ v_{d1}-v_{1} \end{array}\right], \end{array}$$

where *u*_*d*1_: Pixel value of the target on x-axis in Σ_*R*_, *v*_*d*1_: Pixel value of the target on y-axis in Σ_*R*_, *u*_1_: Pixel value of the en-effector on x-axis in Σ_*R*_, *v*_1_: Pixel value of the en-effector on y-axis in Σ_*R*_.

In this study the **W_1_** is given by

(6) $$\begin{array}{l} {\text{W}}_{1}=\left[\begin{array}{cc} \cos\left(\theta_{y1}\right) & 0\\ \sin\left(\theta_{x1}\right)\sin\left(\theta_{y1}\right) & \cos\left(\theta_{x1}\right) \end{array}\right]=\left[\begin{array}{cc} 0.87 & 0\\ 0.43 & 0.50 \end{array}\right], \end{array}$$

with θ_*x*1_ = 60 [deg]: Angle between x-axis of Σ_*R*_ and $\underset{C1}{\sum }$, θ_*y*1_ = 30 [deg]: Angle between x-axis of Σ_*R*_ and $\underset{C1}{\sum }$.

However, **W_1_** is always accompanied by the error from calibration, thus, we approximate **W_1_** to be unit matrix.

**C_1_** is given by

(7) $$\begin{array}{l} {\text{C}}_{1}=\left[\begin{array}{cc} 0 & -1\\ -1 & 0 \end{array}\right]. \end{array}$$

Using the coordinate transformation matrix and the video image obtained from the camera, and from Equation (3), we apply the following control scheme to calculate the target torque;

(8) $$\begin{array}{l} \tau_{\text{d}}\left(\text{t}\right)={\text{K}}_{\text{P}}{{\text{J}}_{\left(\text{q}\text{d}\right)}}^{\text{T}}{\text{C}}_{1}\Delta {\text{U}}_{1}+\int_{0}^{\text{t}}{\text{K}}_{\text{I}}{{\text{J}}_{\left(\text{q}\text{d}\right)}}^{\text{T}}{\text{C}}_{1}\Delta {\text{U}}_{1}\text{d}\delta \end{array}$$

where ${{\text{J}}_{\left(\text{qd}\right)}}^{\text{T}}$ is a transposed Jacobian (Equation 2) which is approximated by the one close to the target position and kept constant throughout the reaching motion. **K**_**P**_ is a gain factor for proportional control, and **K**_**I**_ for integral control.

As a next step, let us calculate the control law based on the robotic coordinate, Σ_*R*_. Using the coordinate transformation from the camera to the work space coordinate system, Equations (4) and (8) becomes

(9) $$\begin{array}{l} \tau_{\text{d}}(\text{t})={\text{K}}_{\text{P}}{\tilde{\text{J}}}^{\text{T}}\Delta \text{X}+\int_{0}^{t}{\text{K}}_{\text{I}}{\tilde{\text{J}}}^{\text{T}}\Delta \text{X}\text{d}\delta \end{array}$$

where ${\tilde{\text{J}}}^{\text{T}}$ has been modified as follows,

(10) $$\begin{array}{l} {\tilde{\text{J}}}^{\text{T}}={{\text{J}}_{\left(\text{q}\text{d}\right)}}^{\text{T}}{\text{C}}_{1}{{\text{W}}_{1}}^{-1}{{\text{C}}_{1}}^{-1}. \end{array}$$

Note here that ${\tilde{\text{J}}}^{\text{T}}$ is the approximated Jacobian, which contains errors. However, the convergence of Δ**X** is guaranteed as long as there is not so much difference between ${\tilde{\text{J}}}^{\text{T}}$ and ${{\text{J}}_{\left(\text{q}\text{d}\right)}}^{\text{T}}$ (Nishida and Kawamura, 2012). Thus, the proposed control scheme here does not require any encoders to measure the angle of the joints, contributing to the ultra-light weight of the exoskeleton.

In order to output the target pressure to control the soft modules, it is necessary to convert the target torque to the target pressure, which require the detail modeling and the non-linear mapping. This relation should be generally given by **P**_**d**_ = **B**_(**q**)_τ_**d**_. However, in this work, we simply related the desired torque with the desired pressure by **P**_**d**_ = **B**_**0**_**τ**_**d**_. Note here, that this simple relation was made possible due to the positive and negative pressure actuation. We set *B*_0_ = 1, and only use **K**_**P**_ and **K**_**I**_ as the gain parameters to be tuned. The gain parameters will be determined empirically for each experimental condition. The schematic summary of the control scheme is given in Figure 2. As experimental results, we will show the trajectories of the end-effector in the scale of [mm], using Δ**U**.

### 3.3. Camera coordinate to record the trajectories of the end-effector

In this section, we explain how we set up the camera coordinate to record the trajectories of the end-effector in Figure 1. While performing the experiments, another camera located above the 2D work plane records the trajectories of the end-effector. Based on the robot coordinate, Σ_*R*_, the relative distance between the target and the end-effector, Δ**X** is given by

(11) $$\begin{array}{l} \Delta \text{X}={\text{C}}_{2}{\text{W}}_{2}\Delta {\text{U}}_{2}, \end{array}$$

where **C_2_**: Coordinate direction transformation matrix from Σ_*C*2_ to Σ_*R*_, **W_2_**: Coordinate rotation transformation matrix from Σ_*C*2_ to Σ_*R*_.

Δ**U_2_** is the relative distance between the target position and the end-effector based on Σ_*C*2_ in Figure 1 given by

(12) $$\begin{array}{l} \Delta {\text{U}}_{2}=\left[\begin{array}{c} u_{d2}-u_{2}\\ v_{d2}-v_{2} \end{array}\right]\;, \end{array}$$

with *u*_*d*2_: Pixel value of the target position on x-axis, *v*_*d*2_: Pixel value of the target position on y-axis, *u*_2_: Pixel value of the end-effector position on x-axis, *v*_2_: Pixel value of the end-effector position on y-axis.

In this study, **W_2_** is calculated by setting the camera on θ_*x*2_ = 0 [deg], θ_*y*2_ = 0 [deg].

(13) $$\begin{array}{l} {\text{W}}_{2}=\left[\begin{array}{cc} \cos\left(\theta_{y2}\right) & 0\\ \sin\left(\theta_{x2}\right)\sin\left(\theta_{y2}\right) & \cos\left(\theta_{x2}\right) \end{array}\right]\;=\left[\begin{array}{cc} 1 & 0\\ 0 & 1 \end{array}\right]\;, \end{array}$$

with θ_*x*2_: Angle between x-axis of Σ_*R*_ and Σ_*C*2_, θ_*y*2_: Angle between y-axis of Σ_*R*_ and Σ_*C*2_.

And **C_2_** is given by

(14) $$\begin{array}{l} {\text{C}}_{2}=\left[\begin{array}{cc} -1 & 0\\ 0 & 1 \end{array}\right]. \end{array}$$

### 3.4. Production of soft actuators and exoskeleton

**Figure 3:**
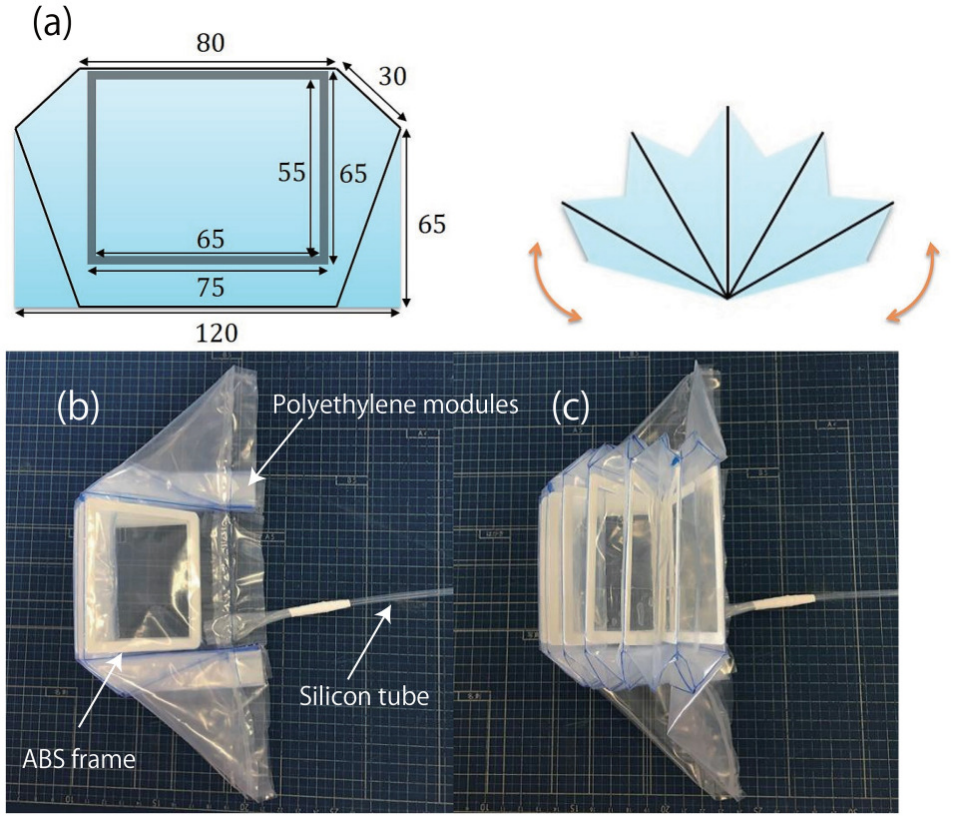
Polyethylene soft modules developed for the active joints. **(a)** Design of the soft modules. **(b)** Soft module without actuation. **(c)** Inflated soft module. The plastic modules were inflated by pneumatic regulators through silicon tubes. The ABS frames were used to maintain the each cell structure when deflating the modules by applying negative pressure.

The polyethylene soft modules developed for the active joints were designed to be attached to the joints (*q*_1_ and *q*_4_ in Figure 1D), and have multi-cells with one end glued together by thermal adhesion (Figure 3). The ABS frames were used to maintain the structure of each cell, when deflating the modules by applying negative pressure.

The inflatable actuator was pressurized by pneumatic actuators through silicon tubes, and as the individual cells start to push against each other, the whole module produces the torque in the fan-shaped manner as a function of pressure. Also, the modules could produce negative torque by being deflated. The pressure could be as low as −30 [kPa] and range up to 30 [kPa]. This range of pressure was sufficient to actuate the exoskeleton carrying the weight of upper limb of participants.

The forces generated by the soft modules are distributed along the entire length of *L*_1_, *L*_3_, and *L*_4_, providing the torque to rotate the shoulder and elbow joint respectively as in Figure 1E. Note here, that reaching motion can be only realized in the two dimensional plane defined by the kinematic structure in Figure 1E. The ABS parts of exoskeleton were printed by a 3D printer. The parts for the parallel link structures were assembled, using the ABS made bolts.

#### 3.4.1. Development of control circuits

**Figure 4:**
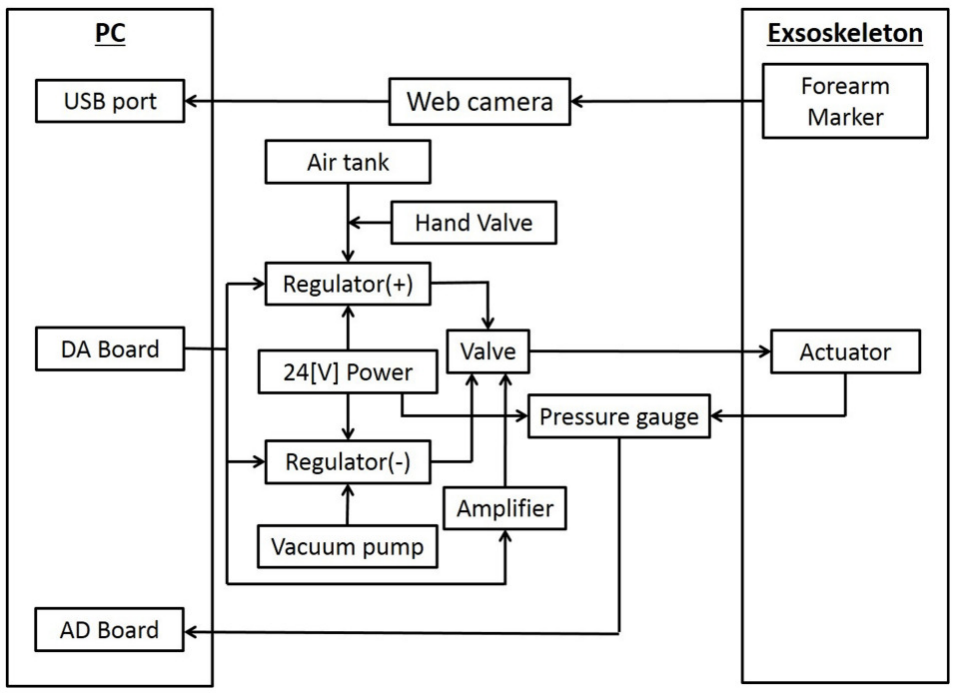
Control Diagram. The vision-based control law was used to minimize the relative position between the target and the tracer. The pressure sensors were placed, and AD board was used to transmit the signals to the PC. The positive pressure regulator and the negative pressure regulator was connected to each soft actuator through the flow regulator valve which switched the pressure between positive and negative. The positive pressure regulator was connected to the tank with the compressor and the negative pressure tank to the vacuum pump.

The control diagram is given in Figure 4. We used a USB camera (logicool HD WEBCAM C525) located above the shoulder of exoskeleton for the visual feedback control to calculate the relative distance between the target and the end-effector. The marker was attached to the forearm to represent the hand position. The visual processing was programmed in C++ (Microsoft Visual Studio 2013). The control law in Equation (8) outputs the desired pressure to actuate the inflatable plastic modules. The desired pressure was transmitted to the regulator which controlled the valves of the air flow via DA board.

In order to measure the pressure at the vicinity of soft modules, the pressure sensors (SMC ISE10-M5-C) were placed, and AD board (Contec AI-1616LI-PE) was used to transmit the signals to the PC. The positive pressure regulator (SMC ITV2050-212BS) and the negative pressure regulator (SMC ITV20290-212BS5) was connected to each soft actuator through the flow regulator valve (SMC VEF3121-1) which switched the pressure between positive and negative. The positive pressure regulator was connected to the tank with the compressor (IWATA TFP04B-10 C) and the negative pressure tank to the vacuum pump (IWATA SCROLL VACUUM PUMP).

## 4. Torque characteristics of soft actuators

**Figure 5:**
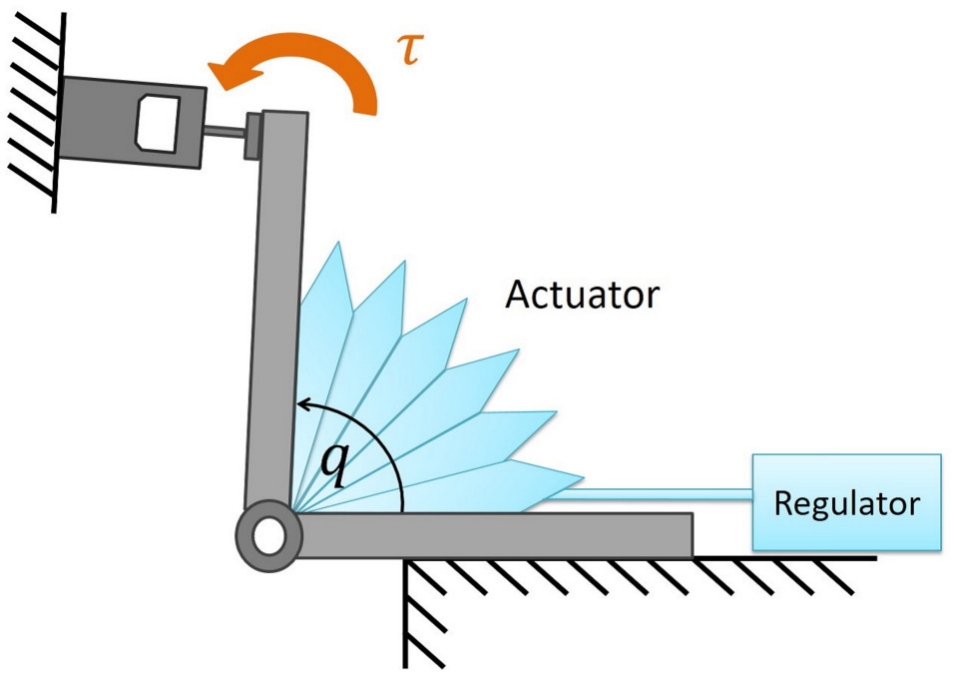
Experimental setup to measure the torque produced by the soft actuator. By changing the angle of the joint, the torque was measured as a function of angle.

The torque characteristics of soft actuators, torque as a function of the joint angle, was investigated using the experimental setup shown in Figure 5. We performed the torque measurement of the soft modules in the following procedure;

1. Fix the angle of the arm in Figure 5.
2. Pressurize the soft module in Figure 5.
3. Measure the force in the force gauge after 5 s from the moment when the force exhibited its highest peak.
4. Go back to the procedure 1 for the next angle.

**Figure 6:**
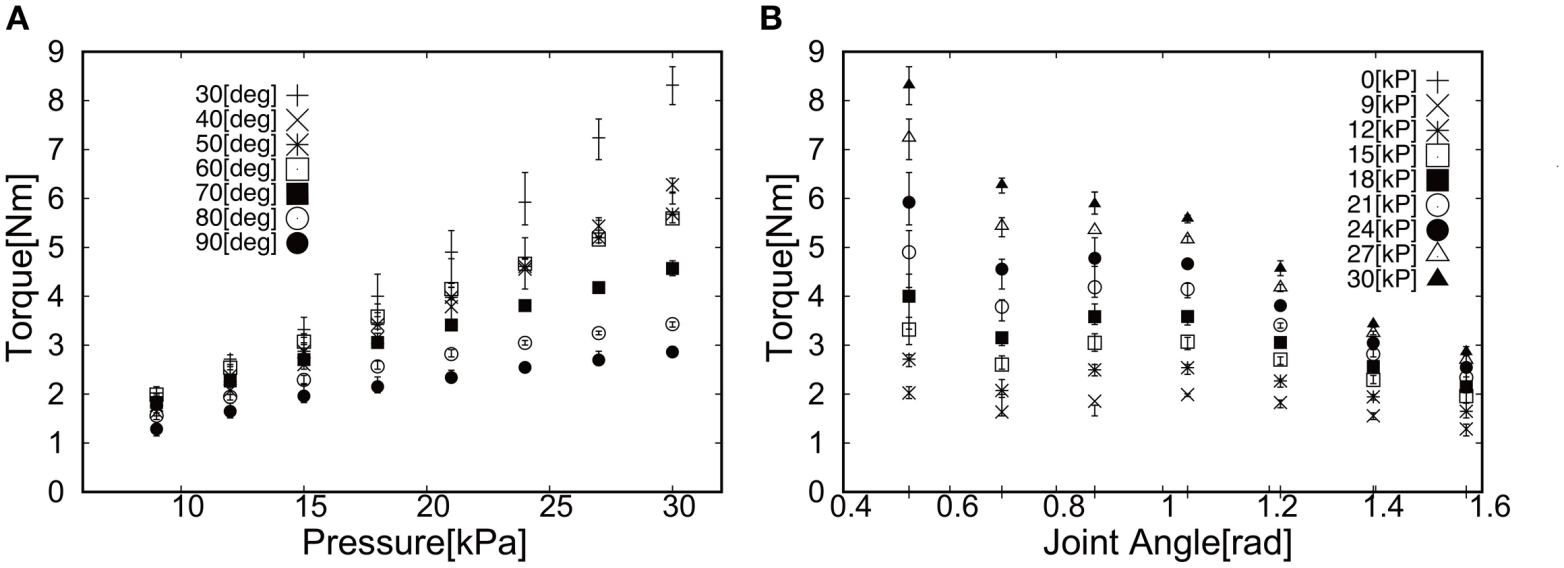
Torque as a function of **(A)** Pressure and **(B)** Joint angle when applied positive pressure.

**Figure 7:**
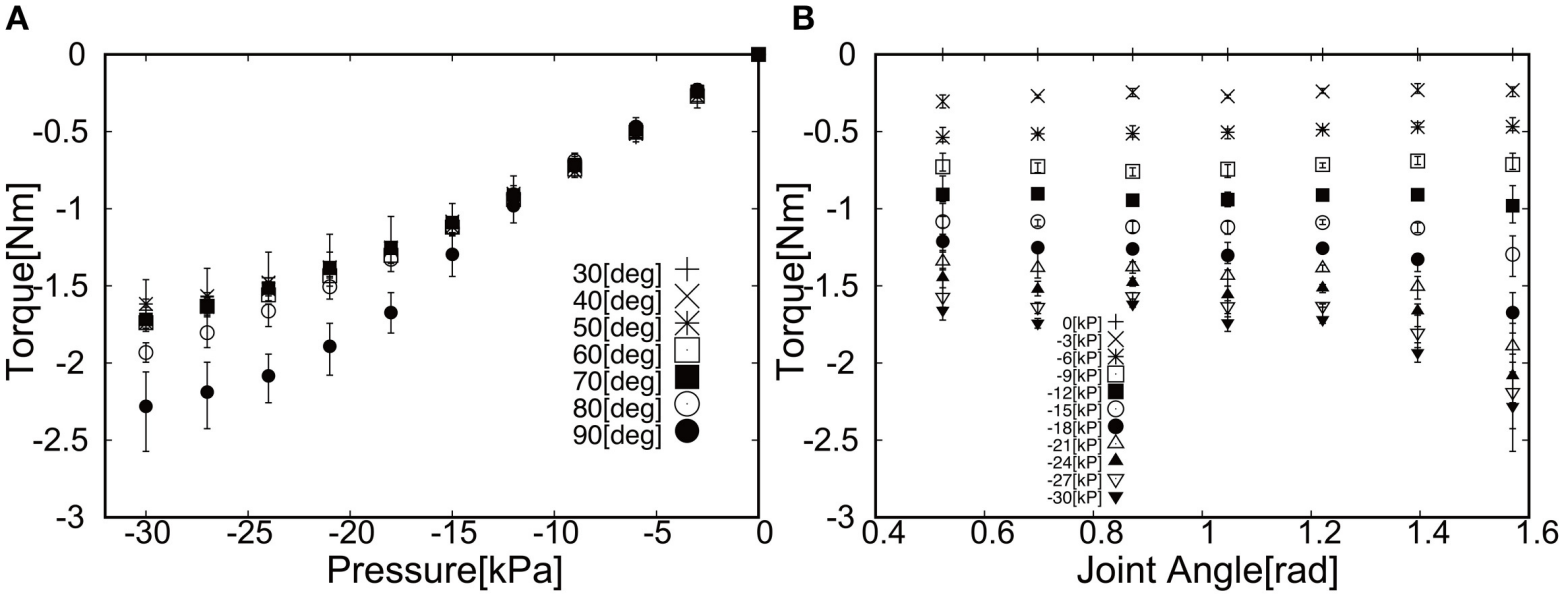
Torque as a function of **(A)** Pressure and **(B)** Joint angle when applied negative pressure.

Thus, in Figures 6, 7 we measured the torque produced by the soft module at the equilibrium state by fixing the angle of the joint in Figure 5. We characterized the functional properties of the soft modules. The torque was measured as a function of (1) pressure in the range from −30 to 30 [kPa] at the incremental step of 3.0 [kPa]; and (2) an angle when the soft module was actuating the rotational joint with two rigid arms (Figure 5).

Figure 6 shows the torque as a function of (a) Pressure and (b) Joint angle. The general trend can be observed; (1) the torque increased as a function of pressure and (2) the torque decreased as a function of angle. For the case of applying the negative pressure to the soft module, Figure 7 shows the torque as a function of (a) Pressure and (b) Joint angle. The general trend can be noted; (1) the torque increased as a function of pressure and (2) the torque increased as a function of an angle.

We expect that the largest hysteresis when switching the pressure from positive to negative, as it takes a certain time for the air flow in pipes and plastic modules to relax to the steady state. However, we aim at the upper limb assistance in slow motion, thus, air flow would be equilibrated throughout the actuation.

## 5. Functional properties of the exoskeleton actuated by soft modules (EASoftM)

**Figure 8:**
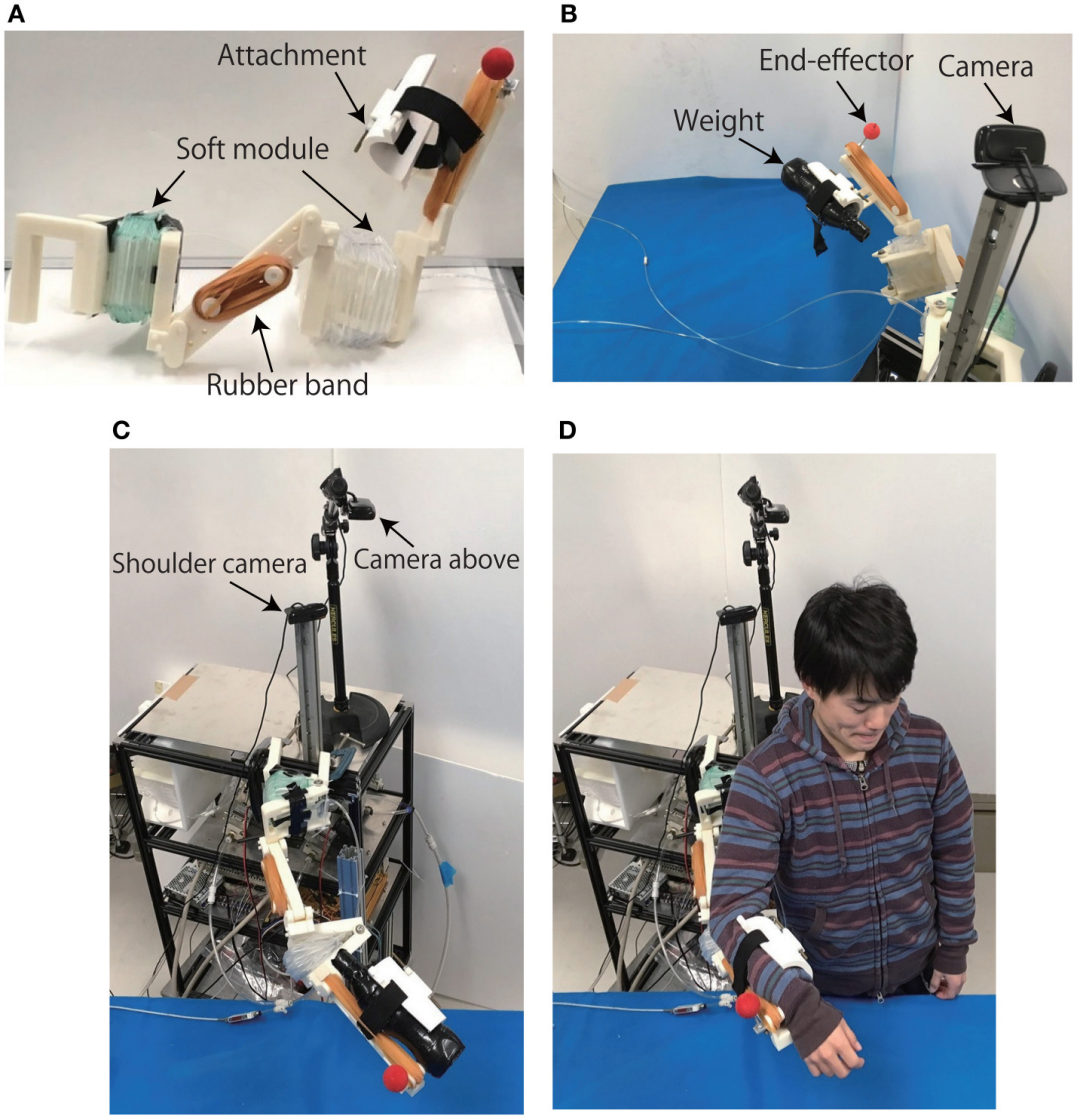
Produced Exoskeleton Actuated by Soft Modules and experimental setups. **(A)** Produced exoskeleton structure with passive and active joints. **(B)** Experimental setup to test the functional properties of the system. The end-effector was attached to the end-point of the links, and the weight of 1.0 [Kg] was attached to the attachment. The camera was located on the shoulder position to apply the visual feedback control. **(C)** Experimental setup of the exoskeleton with the weight on the attachment. **(D)** Experimental setup with the participant (with permission from the participant).

The developed Exoskeleton Actuated by Soft Modules is shown in Figure 8. The rubber bands were attached to the parallel structures of the exoskeleton in order to compensate the gravity, and the soft modules were attached in-between two plastic plates on shoulder and elbow joints. The total weight of the EASoftM was 967 [g], composed of assembled 3D printed arm (724 [g]), soft module (31 [g]), and attachment (181 [g]). The end-effector was attached to the end-point of the links, and the camera was located on the shoulder position to apply the visual feedback control in Figure 1.

In the following sections, we validated the functional properties of the EASoftM, first by putting the weight of 1.0 [kg] on the attachment of the exoskeleton arm, and secondly, by performing the participant experiments as shown in Figure 8D. In both experiments, the relative distance between the end-effector and target, and trajectories in the 2D camera frames were measured as a function of time as well as the pressure applied to the individual soft modules.

### 5.1. Functional properties of the exoskeleton actuated by soft modules (EASoftM) under the weight load

In this section, we demonstrated the functional properties of EASoftM with the weight load of 1.0 [kg] attached to the exoskeleton arm. To validate the motion of the EASoftM for positive and negative pressure actuation, we characterized the motion of the exoskeleton for flexion and extension, respectively, from one degree of freedom (elbow) to two degrees of freedom (elbow and shoulder). Finally, we performed more complex reaching motion which combined the extension and flexion by switching target positions.

#### 5.1.1. 1D elbow motion using the elbow joint of the exoskeleton actuated by soft modules (EASoftM)

##### 5.1.1.1. Flexion motion toward the target (positive pressure actuation)

**Figure 9:**
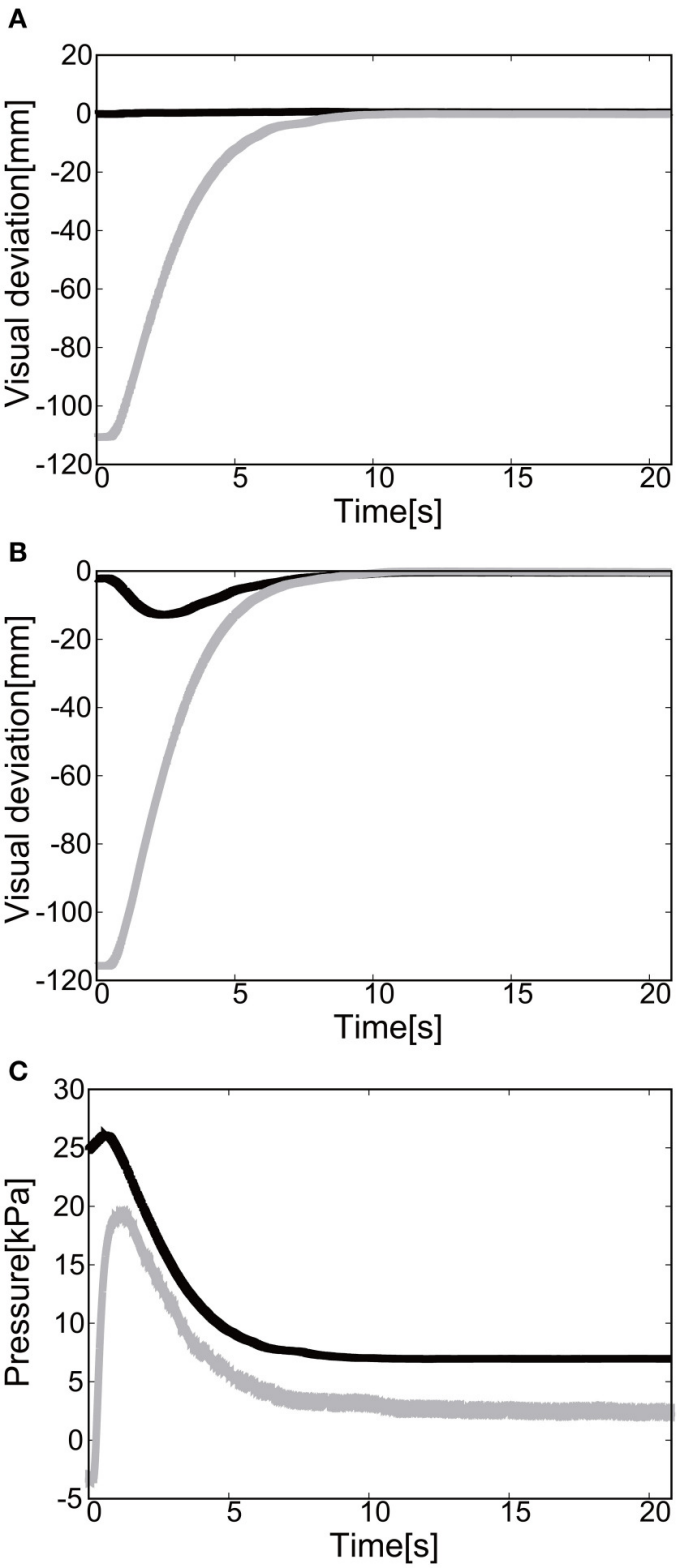
Flexion motion (positive pressure actuation) of the elbow of the Exoskeleton Actuated by Soft Modules. **(A)** Visual deviation (ΔU_1_) *x*(*t*) (Black line) and *y*(*t*) (Gray line) as a function of time, using the camera on the shoulder. **(B)** Visual deviation (ΔU_1_) *x*(*t*) (Black line) and *y*(*t*) (Gray line) as a function of time, using the camera from above. **(C)** The pressure as a function of time. The black line represents the target pressure, and the gray line represents the measured pressure.

For the 1D rotation of the elbow of the EASoftM, the gain parameters were set to *k*_*p*1_ = *k*_*p*2_ = 2.0 and *k*_*i*1_ = *k*_*i*2_ = 0.2 in Equation (8). Figures 9A,B shows the visual deviation (relative distance between the target and the end-effector, Δ*U*_1_ in Equation 5) of *x*(*t*) and *y*(*t*) as a function of time, using the camera on the shoulder. Note here, that this visual deviation was input to the control law in Equation (8) whereas Figure 9B shows the visual deviation (relative distance between the target and the end-effector, Δ*U*_2_ in Equation 12), using the camera from above to record the trajectories of the end-effector. It takes about 12 s to reach the target set at *x* = *y* = 0.

Figure 9C shows the pressure of the soft module as a function of time. Since the relative distance between the target and the tracer is highest at the initial position of the tracer, the pressure profile shows the slow convergence to the target pressure, reflecting the slow pneumatic actuation of the soft modules. Due to the loss of the pressure through the pipelines, the deviation between the target pressure and the measure pressure remained.

**Figure 10:**
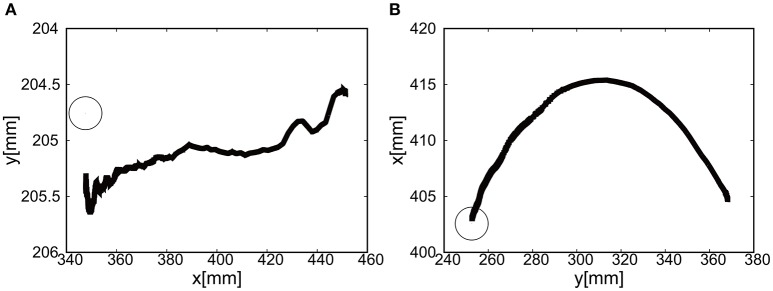
Trajectories of the end-effector in x-y plane (positive pressure actuation), using **(A)** the camera on the shoulder and **(B)** the camera from above. The target is denoted by the circle.

The final visual deviation was △*x* = 0.44 [mm] and △*y* = −0.20 [mm] in Figure 10A, demonstrating the substantial accuracy for reaching motion. This accuracy also proves that the visual-based control as represented in Equation (8) can work efficiently with the approximated kinematics. The trajectory of the tracer attached to the attachment of the exoskeleton shows the circular trajectory as a result of the motion of the elbow rotation in Figure 10B.

##### 5.1.1.2. Extension motion to the target (negative pressure actuation)

**Figure 11:**
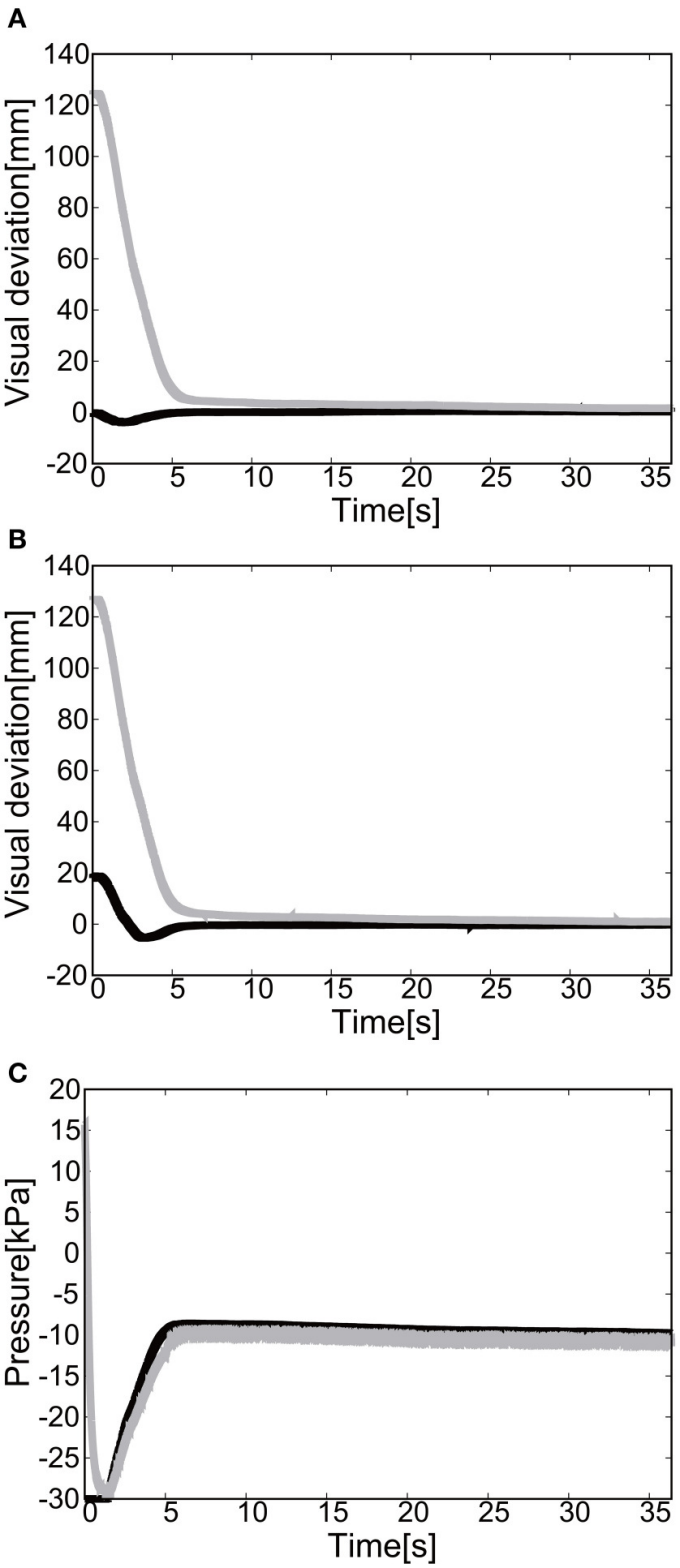
Extension motion (negative pressure actuation) of the elbow of the Exoskeleton Actuated by Soft Modules. **(A)** Visual deviation (ΔU_1_) *x*(*t*) (Black line) and *y*(*t*) (Gray line) as a function of time, using the camera on the shoulder. **(B)** Visual deviation (ΔU_2_) *x*(*t*) (Black line) and *y*(*t*) (Gray line) as a function of time, using the camera from above. **(C)** The pressure as a function of time. The black line represents the target pressure, and the gray line represents the measured pressure.

**Figure 12:**
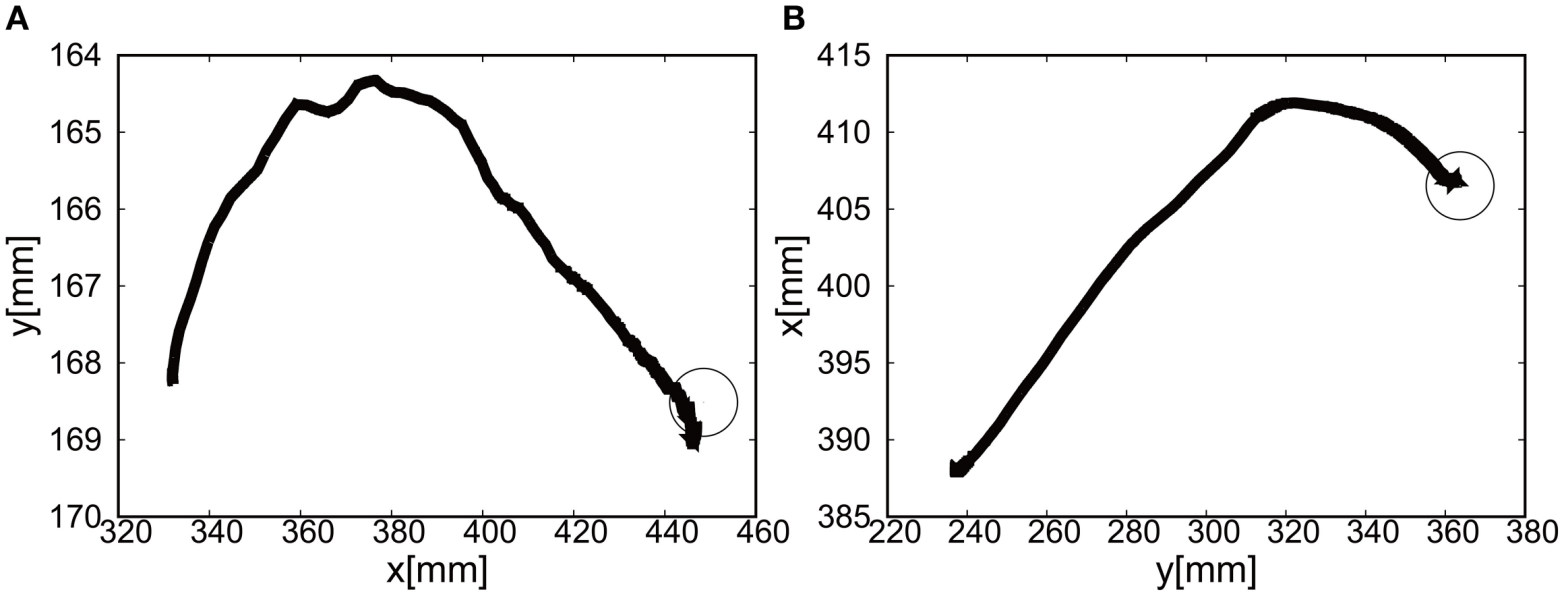
Trajectories of the end-effector in x-y plane (negative pressure actuation), using **(A)** the camera on the shoulder and **(B)** the camera from above. The target is denoted by the circle.

To actuate the soft modules by the negative pressure, the gain parameters were set to *k*_*p*1_ = *k*_*p*2_ = 2.5 and *k*_*i*1_ = *k*_*i*2_ = 0.2 in Equation (8). We could successfully show that extension of the elbow of the EMSoftM was possible by applying the negative pressure as shown in Figure 11. The convergence was shown to the target position (Figures 11A,B) and to the target pressure (Figure 11C). The ABS frame inserted to each plastic cell worked to prevent the collapse of the structure of the cells when deflating air from them. Figure 12 shows the trajectories of the end-effector, demonstrating the extension of the exoskeleton. The final visual deviation was △*x* = 0.30 [mm] and △*y* = 1.65 [mm] in Figure 12A.

#### 5.1.2. 2D motion using the elbow and the shoulder joints of the exoskeleton actuated by soft modules (EASoftM)

As a next step, to validate the 2D motion using the elbow and the shoulder rotation, we performed the experiments using the weight on the attachment under following conditions; (1) Flexion motion to the target, (2) Extension motion to the target, (3) Triangular motion with three targets.

##### 5.1.2.1. Flexion motion to the target (positive pressure actuation)

**Figure 13:**
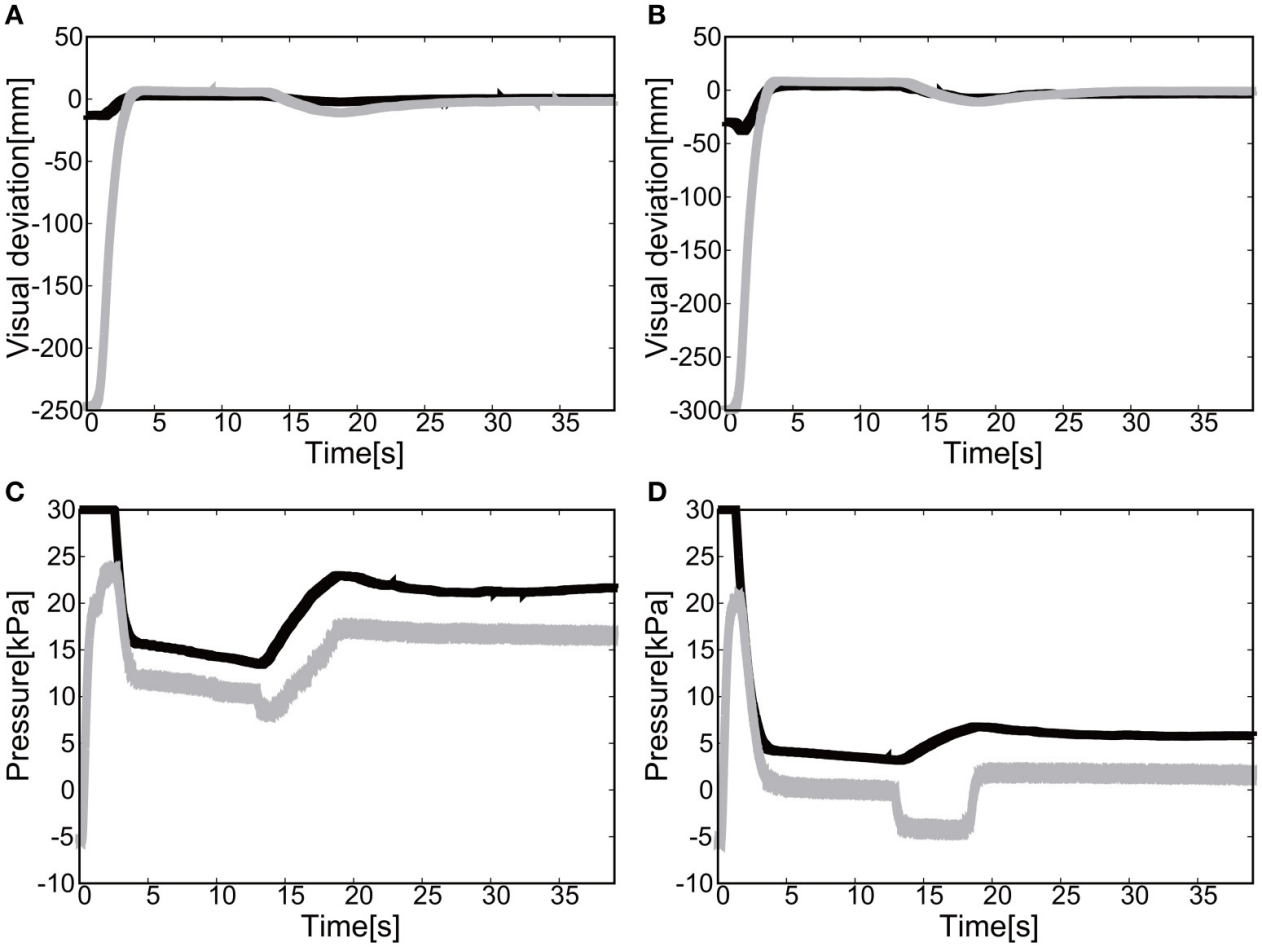
Flexion motion (positive pressure actuation) of the elbow and the shoulder of the Exoskeleton Actuated by Soft Modules. **(A)** Visual deviation (ΔU_1_) *x*(*t*) (Black line) and *y*(*t*) (Gray line) as a function of time, using the camera on the shoulder. **(B)** Visual deviation (ΔU_1_) *x*(*t*) (Black line) and *y*(*t*) (Gray line) as a function of time, using the camera from above. **(C)** The pressure of the shoulder module as a function of time. The black line represents the target pressure, and the gray line represents the measured pressure. **(D)** The pressure of the elbow module as a function of time. The black line represents the target pressure, and the gray line represents the measured pressure.

The gain parameters were set to *k*_*p*1_ = 2.0, *k*_*p*2_ = 2.0 for the elbow joint, and *k*_*p*1_ = 0.2, *k*_*p*2_ = 0.2 for the shoulder in Equation (8). For the case of 2D motion using the elbow and shoulder joints of the EASoftM, it was shown that the reaching motion was also possible as shown in Figures 13A,B.

**Figure 14:**
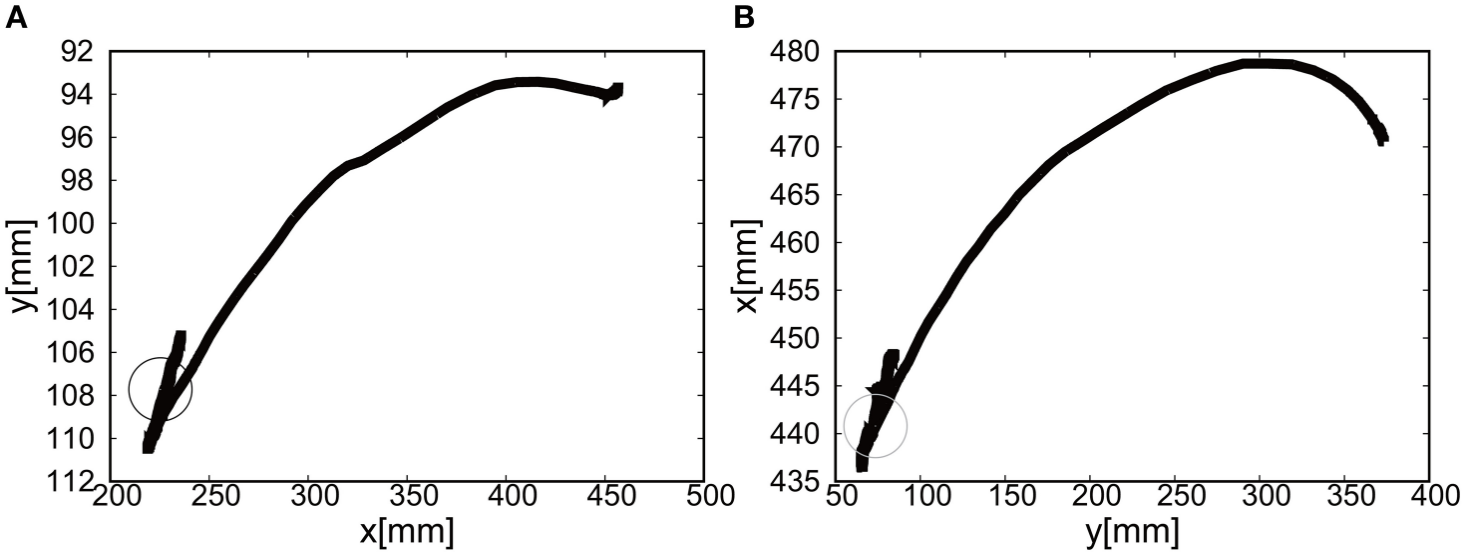
Trajectories of the end-effector (positive pressure actuation) in x-y plane, using **(A)** the camera on the shoulder and **(B)** the camera from above. The target is denoted by the circle.

The drop in pressure around 14 s in Figures 13C,D was due to the change of torque generated by the posture transformation and the gravitational force, as the exoskeleton arm flexed. The non-linear nature of this drop should come from the kinematics of the exoskeleton arm, which is highly non-linear itself. The flow regulator valve controls the constant air flows from the positive and negative regulator, and the pressure within a range of −5.0 and 5.0 [kPa] is outside the precision of the air flow/pressure regulators. However, due to the friction between the ABS frames, this low pressure range will not result in any substantial torque generation. Since the relative distance between the target and the tracer is highest at the initial position of the tracer, the pressure profile shows the slow convergence reflecting the slow pneumatic actuation of the soft modules. The trajectories of the end-effector are shown in Figure 14.

The relative distance to the target was achieved to be △*x* = 0.65 [mm] and △*y* = −1.75 [mm]. This precision is surprising as the control laws in Equation (8) only used the visual information of the relative distance between the target and the end-effector as input to the control law. Note here, that the kinematics of the exoskeleton is only provided as approximation and is kept constant throughout the reaching motion. In the classical control theory, once a target has been defined in the robots work space using its sensors, the necessary final pose to intercept the object must be calculated using some form of inverse kinematics: the explicit transformation of work space coordinates into joint angles. However, the inverse kinematics is very sensitive to error, thus, the visual-based control should be appropriate for control of soft modules.

##### 5.1.2.2. Extension motion to the target (negative pressure actuation)

**Figure 15:**
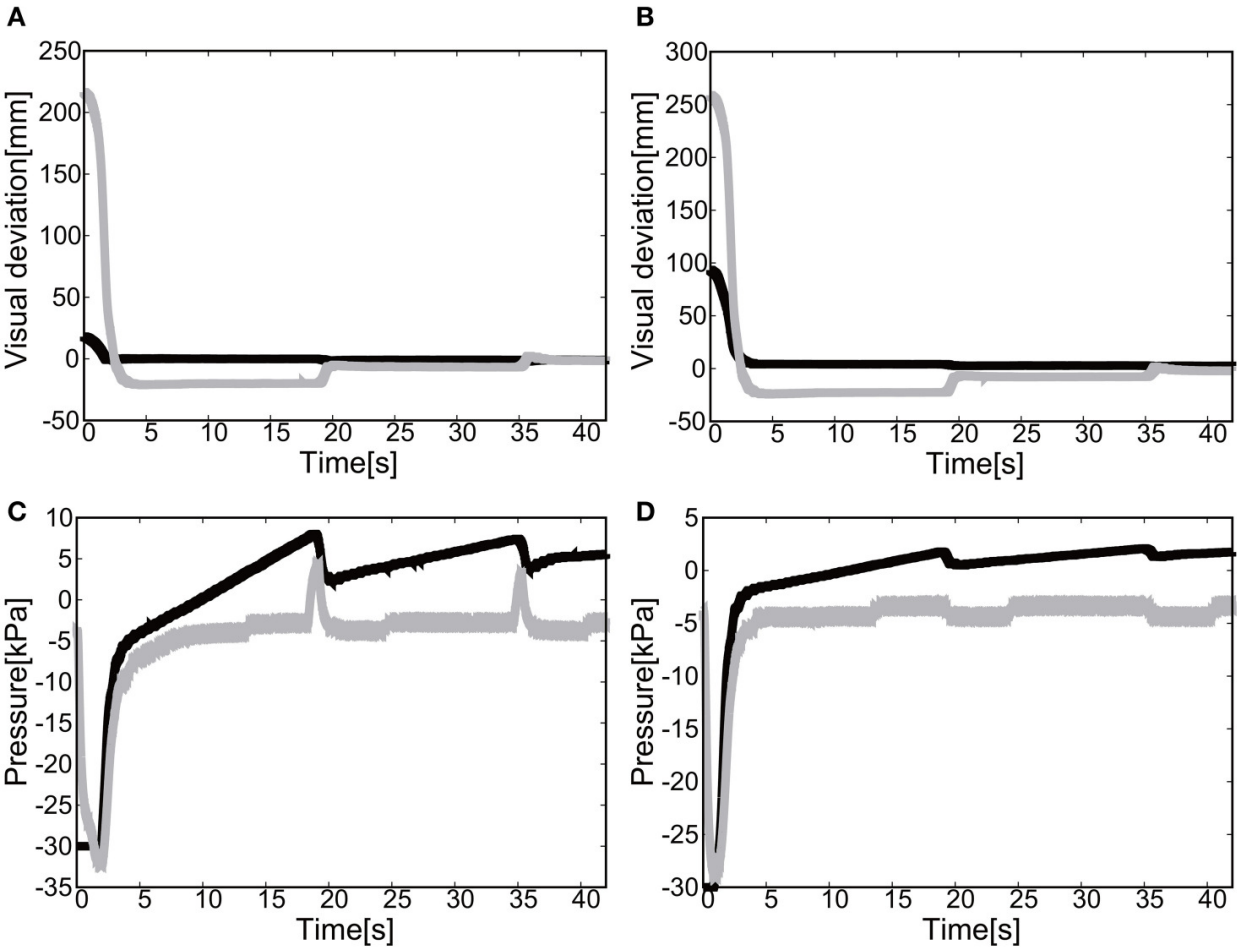
Extension motion (negative pressure actuation) of the elbow and the shoulder of the Exoskeleton Actuated by Soft Modules. **(A)** Visual deviation (ΔU_1_) *x*(*t*) (Black line) and *y*(*t*) (Gray line) as a function of time, using the camera on the shoulder. **(B)** Visual deviation (ΔU_2_) *x*(*t*) (Black line) and *y*(*t*) (Gray line) as a function of time, using the camera from above. **(C)** The pressure of the shoulder module as a function of time. The black line represents the target pressure, and the gray line represents the measured pressure. **(D)** The pressure of the elbow module as a function of time. The black line represents the target pressure, and the gray line represents the measured pressure.

**Figure 16:**
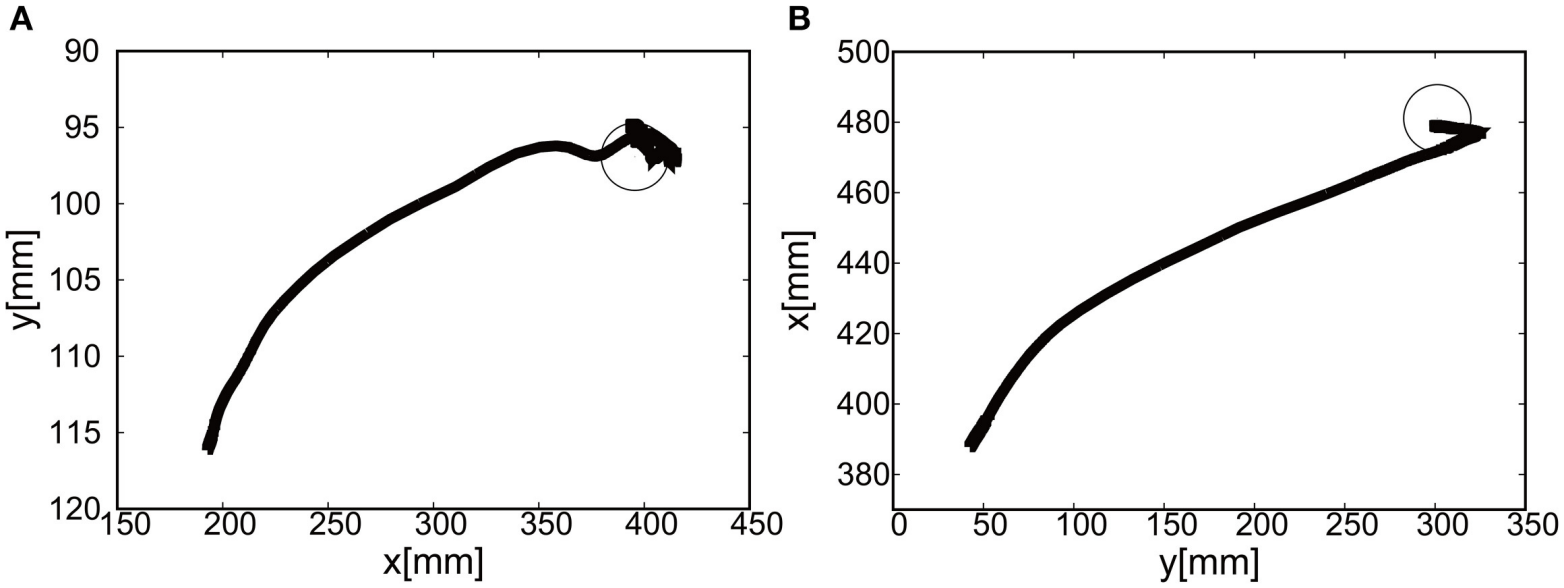
Trajectories of the end-effector in x-y plane (negative pressure actuation), using **(A)** the camera on the shoulder and **(B)** the camera from above. The target is denoted by the circle.

The gain parameters were set to *k*_*p*1_ = 2.0, *k*_*p*2_ = 2.0 for the elbow joint, and *k*_*p*1_ = 0.2, *k*_*p*2_ = 0.2 for the shoulder joint in Equation (8). It was shown that the extension motion is also possible when applying the negative pressure to achieve the flexion motion as shown in Figures 15A,B. Due to the integral control accumulating the visual deviation and the friction between the ABS frames, there were, around 20 and 35 s, two steep changes in values of the measured pressure in Figures 15C,D. Note here that the pressure range between −5.0 and 5.0 [kPa] is out of the controllable range of the flow/pressure regulators in Figure 4. The measured negative pressure in the flow regulation valve is attributed to the constant deflation of the air from the negative pressure regulator. Figure 16 shows the trajectories of the end-effector. The relative distance to the target was achieved to be △*x* = −0.93 [mm] and △*y* = −1.57 [mm].

##### 5.1.2.3. Reaching motion with three target positions

We placed three targets to allow the end-effector to demonstrate the closed loop motion, i.e., the end-effector should come back to its initial place. The target was switched to another target when the relative distance between the target and the end-effector reaches <5.0 [mm]. The gain parameters were set to *k*_*p*1_ = 2.0, *k*_*p*2_ = 2.0 for the elbow joint, and *k*_*p*1_ = 0.2, *k*_*p*2_ = 0.2 for the shoulder joint in Equation (8).

**Figure 17:**
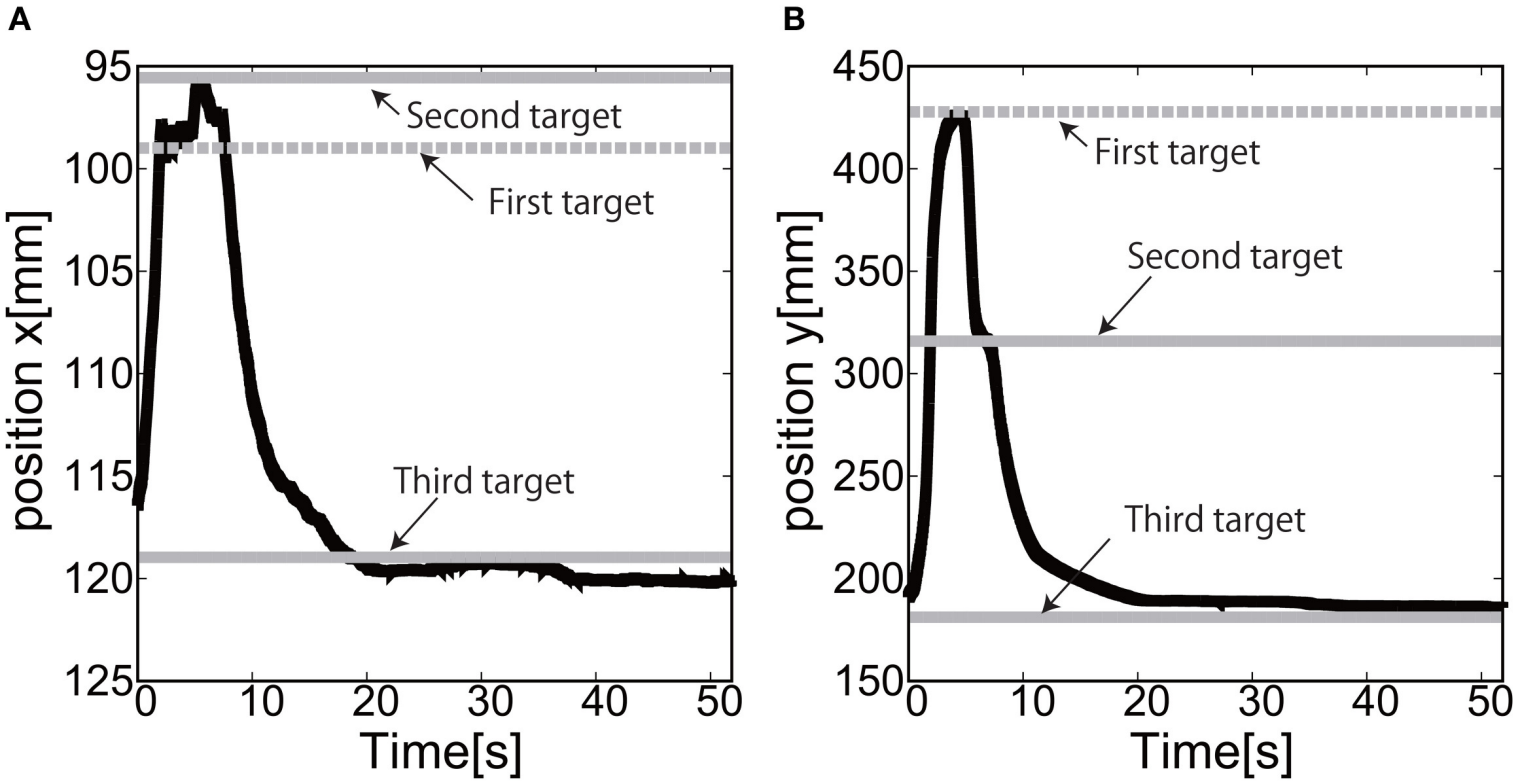
Reaching motion using the elbow and shoulder joint of the Exoskeleton Actuated by Soft Modules, using the camera on the shoulder. **(A)** *x*(*t*) as a function of time. **(B)** *y*(*t*) as a function of time.

**Figure 18:**
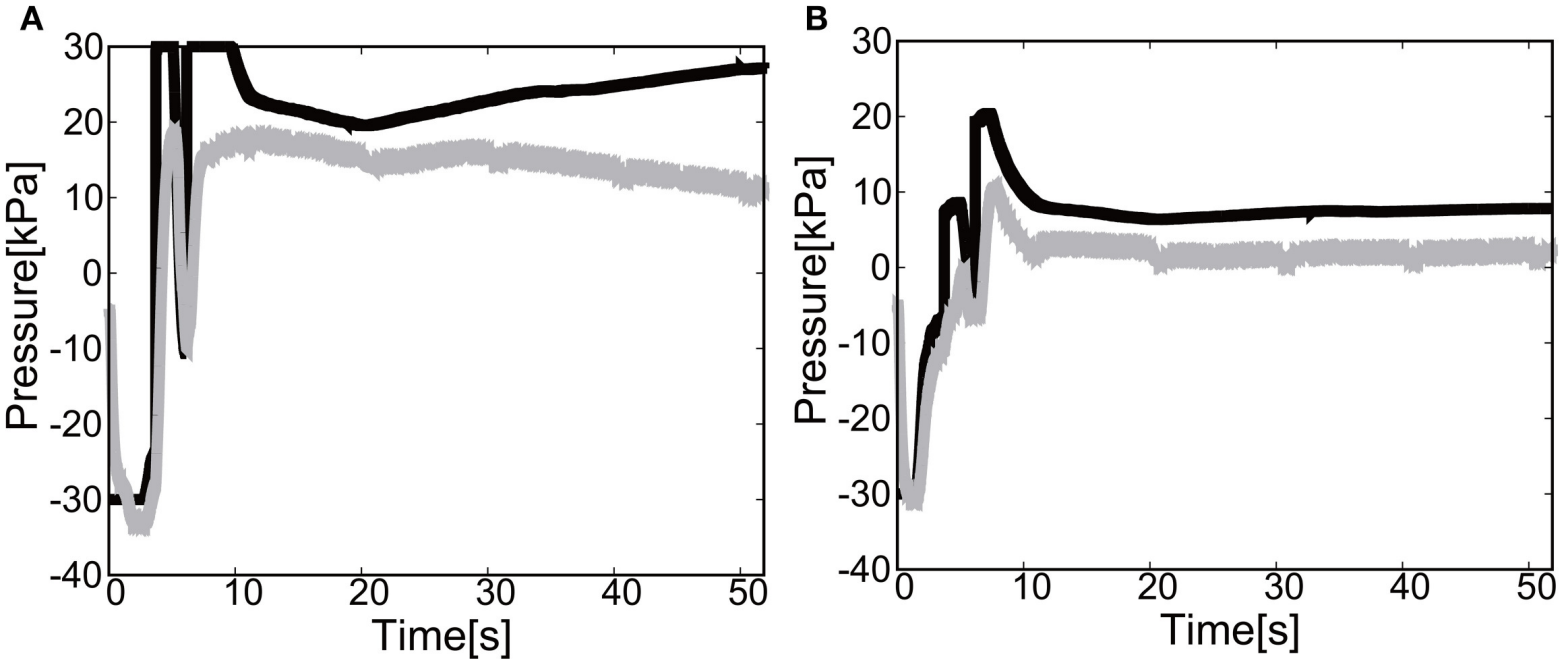
Reaching motion using the elbow and shoulder joint of the Exoskeleton Actuated by Soft Modules, using the camera on the shoulder. The black line represents the target pressure, and the gray line represents the measured pressure. **(A)** The pressure of the shoulder module as a function of time. **(B)** The pressure of the elbow module as a function of time.

Figures 17A,B show the position of the target, *x*(*t*) and *y*(*t*), using the camera on the shoulder. We can clearly observe that the end-point reaches the first target, the second target, and goes back to the initial position where the third target was located. Figure 18 shows the target and measured pressure as a function of time for the shoulder (a) and the elbow (b) joint. When switching the target position, there were corresponding jumps in the target pressure, but note here that the measured pressure followed a smooth curve from the positive to negative pressure around 7 s in Figures 18A,B. It is a promising feature of the soft module used to actuate the exoskeleton to demonstrate the smooth transition for switching the sign of the pressure, leading to the smooth transformation of motion from flexion to extension.

**Figure 19:**
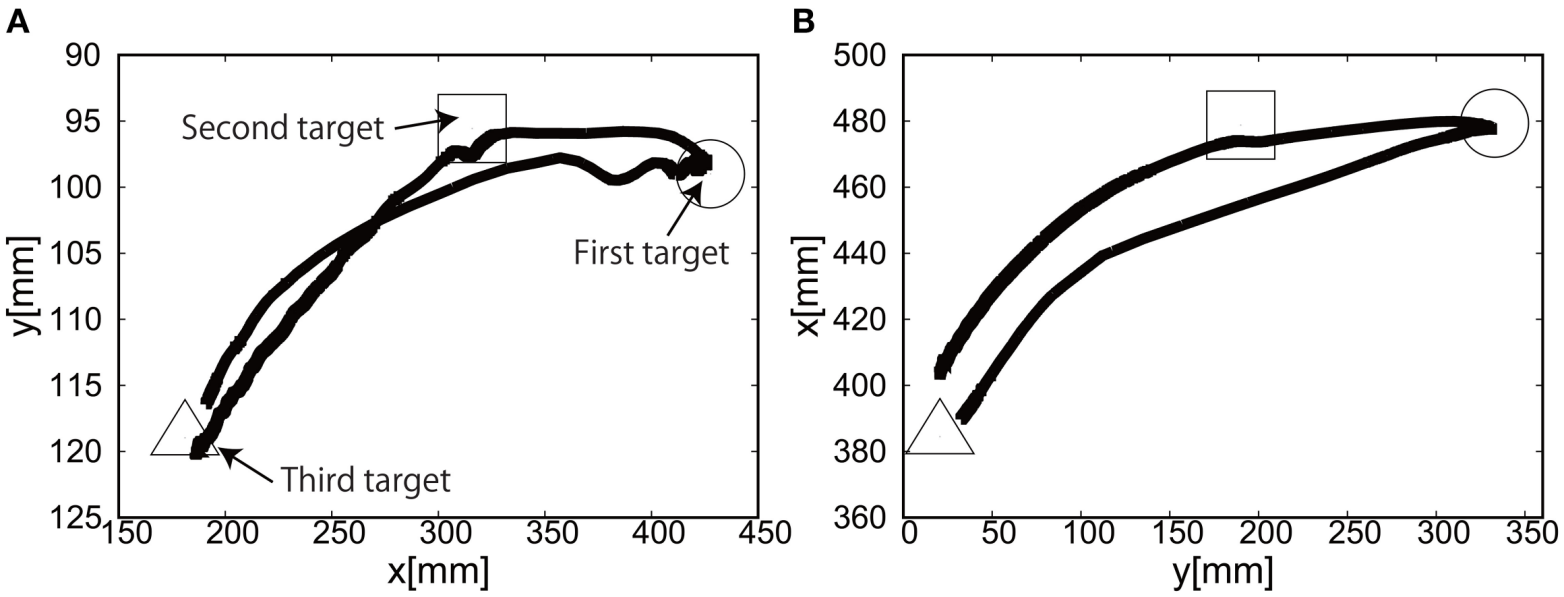
Trajectories of the end-effector in x-y plane, using **(A)** the camera on the shoulder and **(B)** the camera from above. The first target is denoted by circle, the second target by square, and the third target by triangle.

For the case of reaching motion by switching three targets sequentially, it was shown that the reaching motion could demonstrate the closed loop motion as shown in Figure 19, even though there was some overshoot in the relative distance and the measured pressure as a function of time when switching the targets.

### 5.2. Functional properties of the exoskeleton actuated by soft modules (EASoftM) validated by participant experiments

To evaluate the functionality of EASoftM in providing assistance with a wide range of reaching motion and precision to reach a target, we performed the experiments with one participant. The ethical approval was permitted by the ethical committee of Ritsumeikan University (BKC-medical-2016-051). Figure 8D shows the Exoskeleton system attached to the wrist of the participant. The equilibrium point of the upper limb was shifted to match the desired work space appropriate for reaching motion. The visual process using the camera on the shoulder could successfully monitor the position of the tracer to apply the visual control law (Equation 8).

#### 5.2.1. Flexion motion to the target (positive pressure actuation)

**Figure 20:**
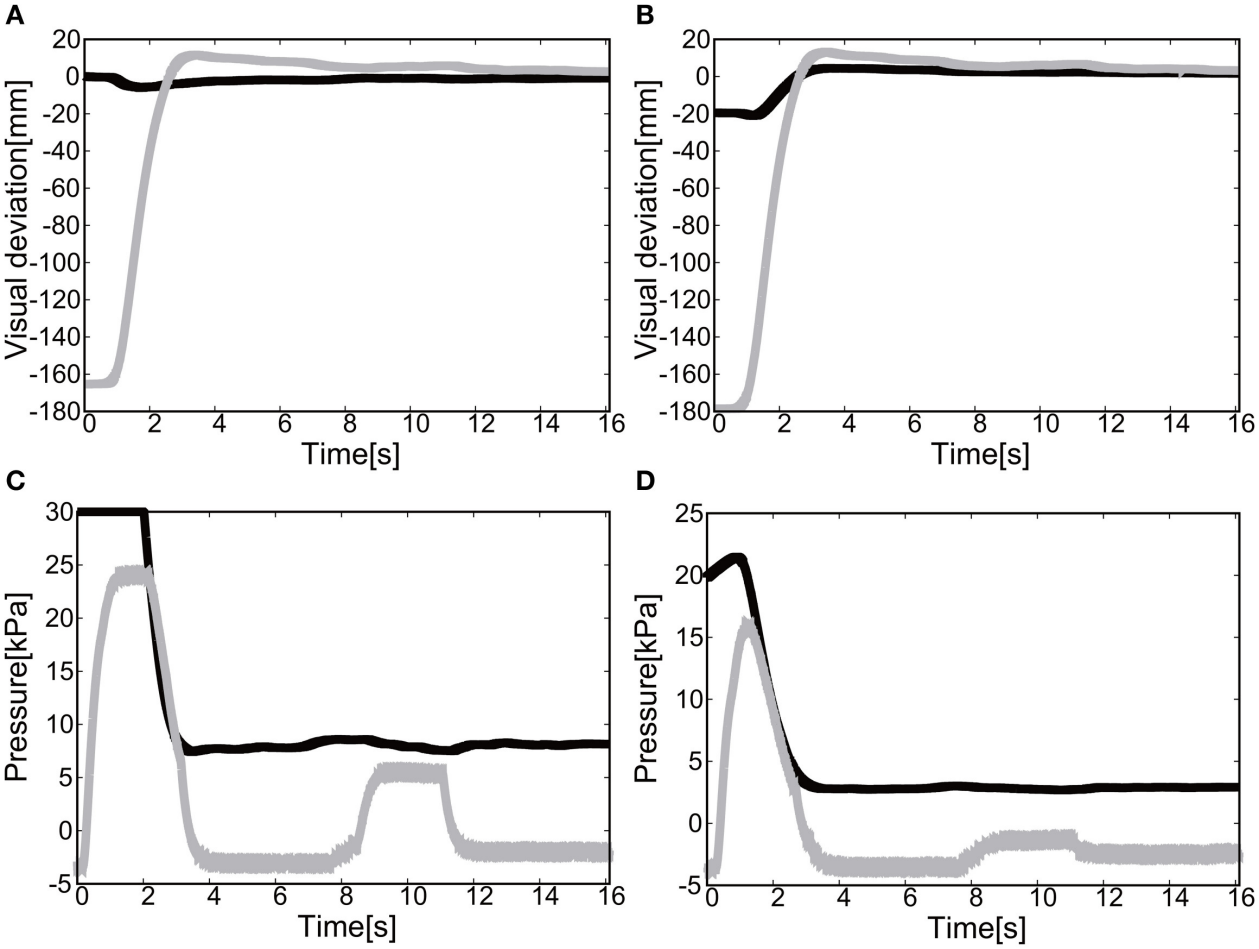
Flexion motion (positive pressure actuation) of the elbow and the shoulder of the Exoskeleton with the participant. **(A)** Visual deviation (ΔU_1_) *x*(*t*) (Black line) and *y*(*t*) (Gray line) as a function of time, using the camera on the shoulder. **(B)** Visual deviation (ΔU_1_) *x*(*t*) (Black line) and *y*(*t*) (Gray line) as a function of time, using the camera from above. **(C)** The pressure of the shoulder module as a function of time. The black line represents the target pressure, and the gray line represents the measured pressure. **(D)** The pressure of the elbow module as a function of time. The black line represents the target pressure, and the gray line represents the measured pressure.

The gain parameters were set to *k*_*p*1_ = 2.0, *k*_*p*2_ = 2.0 for the elbow joint, and *k*_*p*1_ = 0.2, *k*_*p*2_ = 0.2 for the shoulder in Equation (8). For the case of 2D motion using the elbow and shoulder joints of the EASoftM, it was shown that the reaching motion is also possible, as shown in Figures 20A,B.

**Figure 21:**
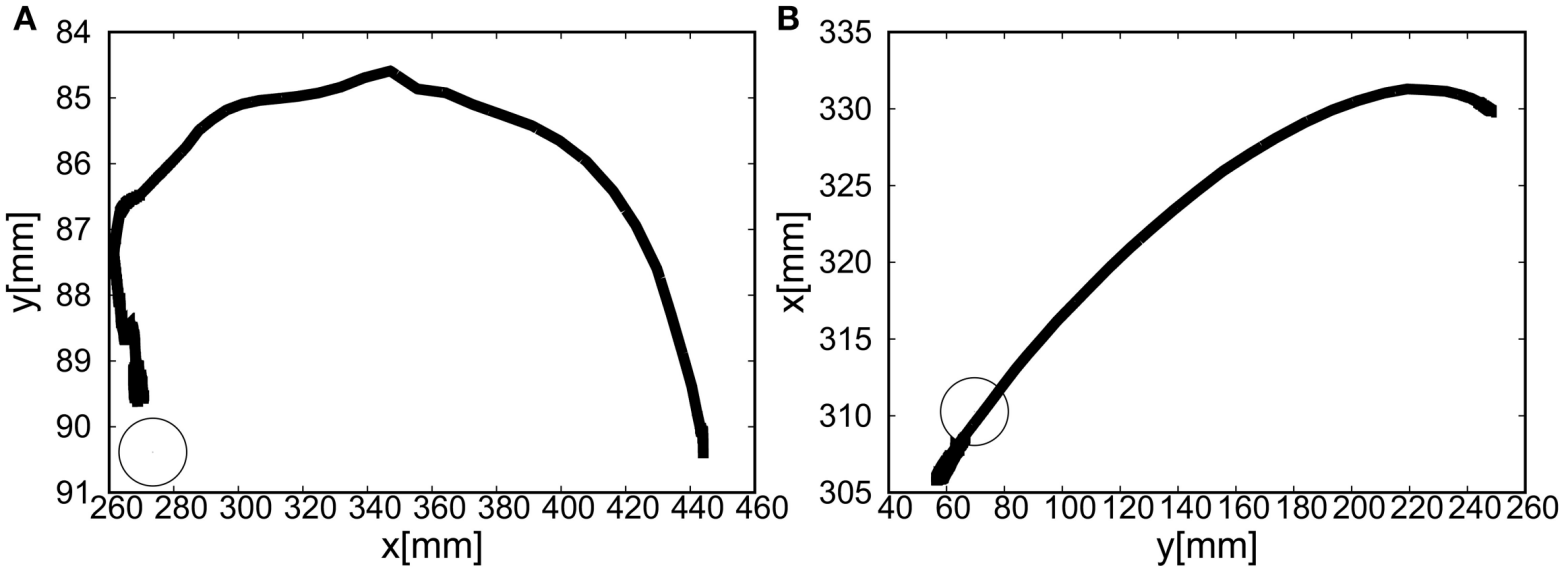
Trajectories of the end-effector (positive pressure actuation) in x-y plane (with the participant), using **(A)** the camera on the shoulder and **(B)** the camera from above. The target is denoted by the circle.

The sudden rise in pressure around 8 s in Figures 20C,D was due to the configuration changes of the soft modules under the friction. However, this type of fluctuation within the low pressure range does not produce enough torque to affect the rotation of the joints Since the relative distance between the target and the tracer is highest at the initial position of the tracer, the pressure profile shows the slow convergence reflecting the slow pneumatic actuation of the soft modules. The trajectories of the end-effector are shown in Figure 21. As the exoskeleton itself was designed based on the anatomical nature of human body, the two link motion in parallel to the human upper limb was smooth. The relative distance to the target was achieved to be △*x* = −0.89 [mm] and △ *y* = 2.66 [mm].

#### 5.2.2. Extension motion to the target (negative pressure actuation)

**Figure 22:**
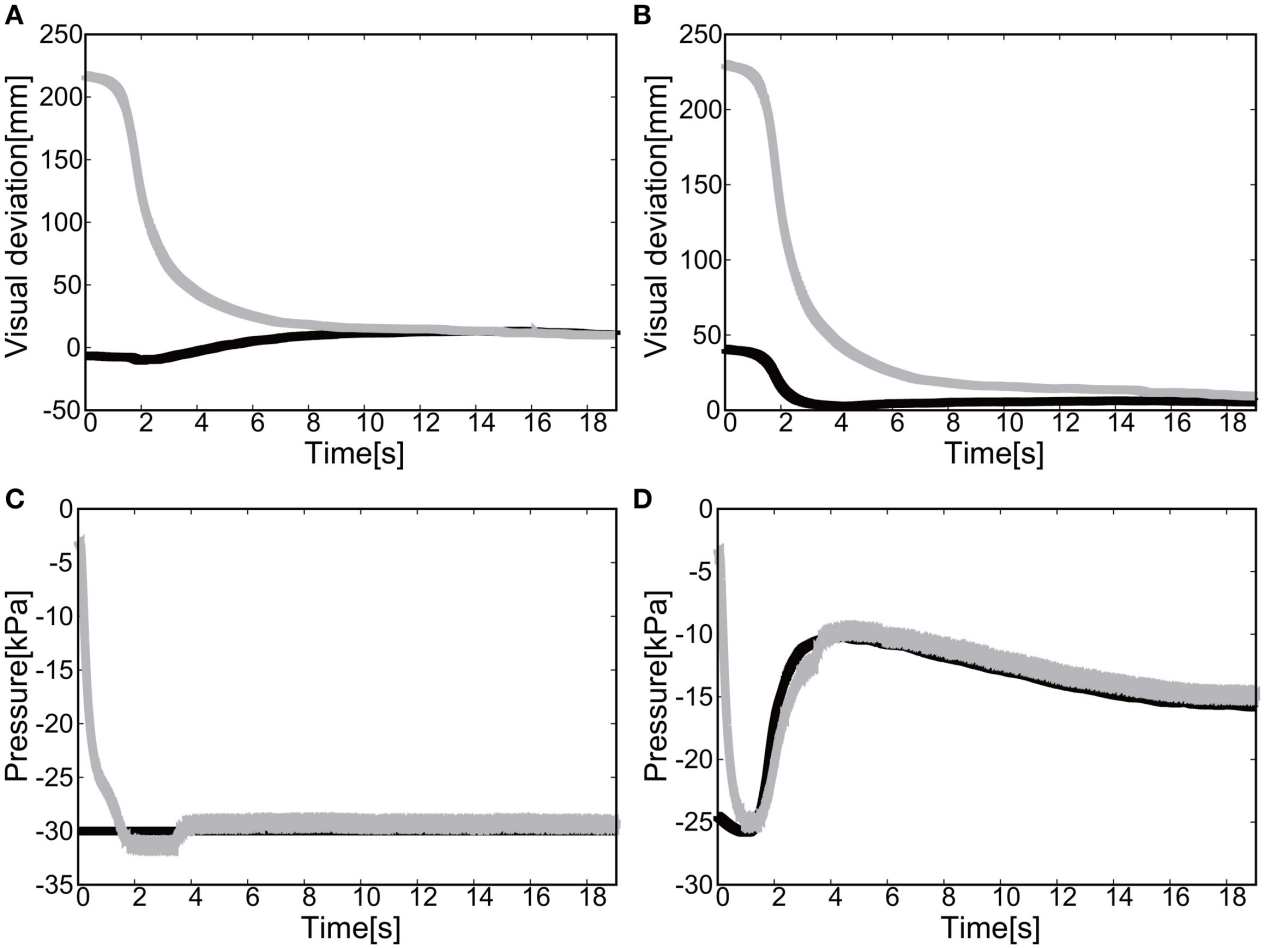
Extension motion (negative pressure actuation) of the elbow and the shoulder of the Exoskeleton with the participant. **(A)** Visual deviation (ΔU_1_) *x*(*t*) (Black line) and *y*(*t*) (Gray line) as a function of time, using the camera on the shoulder. **(B)** Visual deviation (ΔU_2_) *x*(*t*) (Black line) and *y*(*t*) (Gray line) as a function of time, using the camera from above. **(C)** The pressure of the shoulder module as a function of time. The black line represents the target pressure, and the gray line represents the measured pressure. **(D)** The pressure of the elbow module as a function of time. The black line represents the target pressure, and the gray line represents the measured pressure.

**Figure 23:**
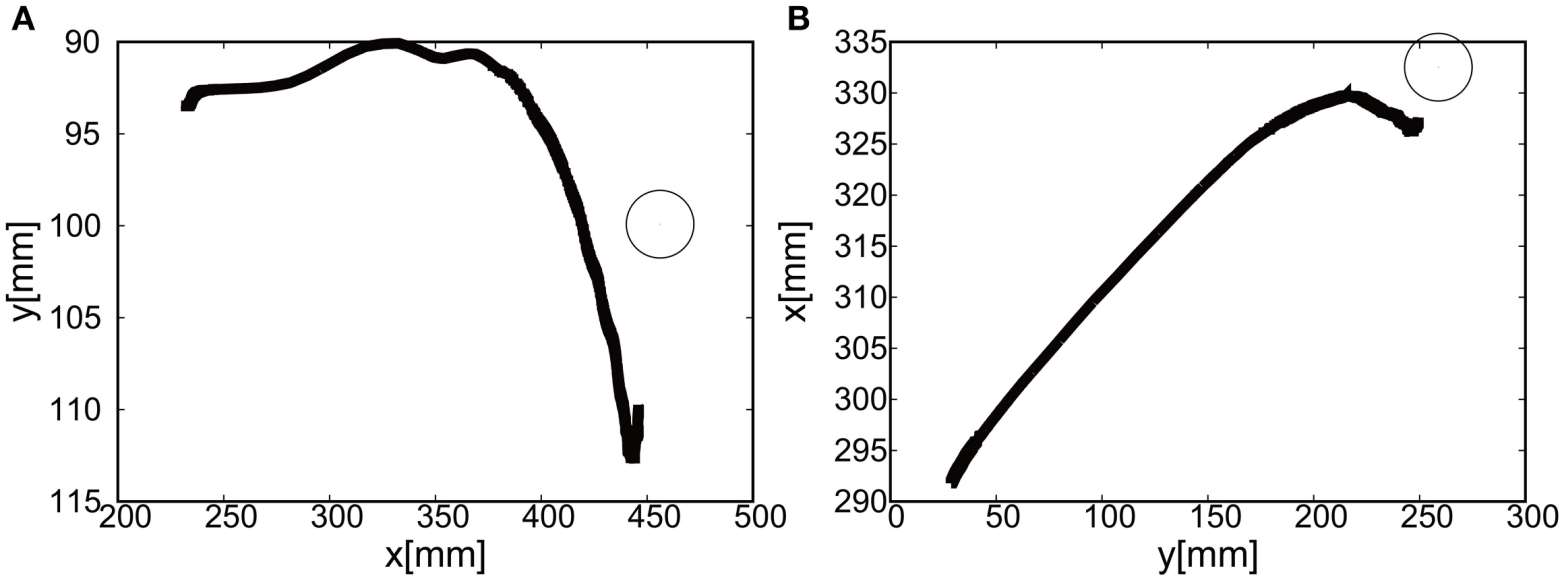
Trajectories of the end-effector (negative pressure actuation) in x-y plane (with the participant), using **(A)** the camera on the shoulder and **(B)** the camera from above. The target is denoted by the circle.

The gain parameters were set to *k*_*p*1_ = 2.0, *k*_*p*2_ = 2.0 for the elbow joint, and *k*_*p*1_ = 0.2, *k*_*p*2_ = 0.2 for the shoulder joint in Equation (8). It was shown that the extension motion is also possible when applying the negative pressure to achieve the flexion motion as shown in Figures 22A,B. Figures 22C,D show the pressure of the shoulder and elbow module respectively as a function of time. After 3 s from the initial targets, their pressure converged to the target pressure. Figure 23 shows the trajectories of the end-effector. The relative distance to the target was achieved to be △*x* = 10.12 [mm] and △ *y* = 9.92 [mm].

#### 5.2.3. Reaching motion with three target positions

By switching three targets sequentially, we demonstrated the closed loop motion, i.e., the end-effector should come back to its initial place. The target was switched to another target when the relative distance between the target and the end-effector reached <5.0 [mm]. The gain parameters were set to *k*_*p*1_ = 2.20, *k*_*p*2_ = 2.20 for the elbow joint, and *k*_*p*1_ = 0.50, *k*_*p*2_ = 0.50 for the shoulder joint in Equation (8).

**Figure 24:**
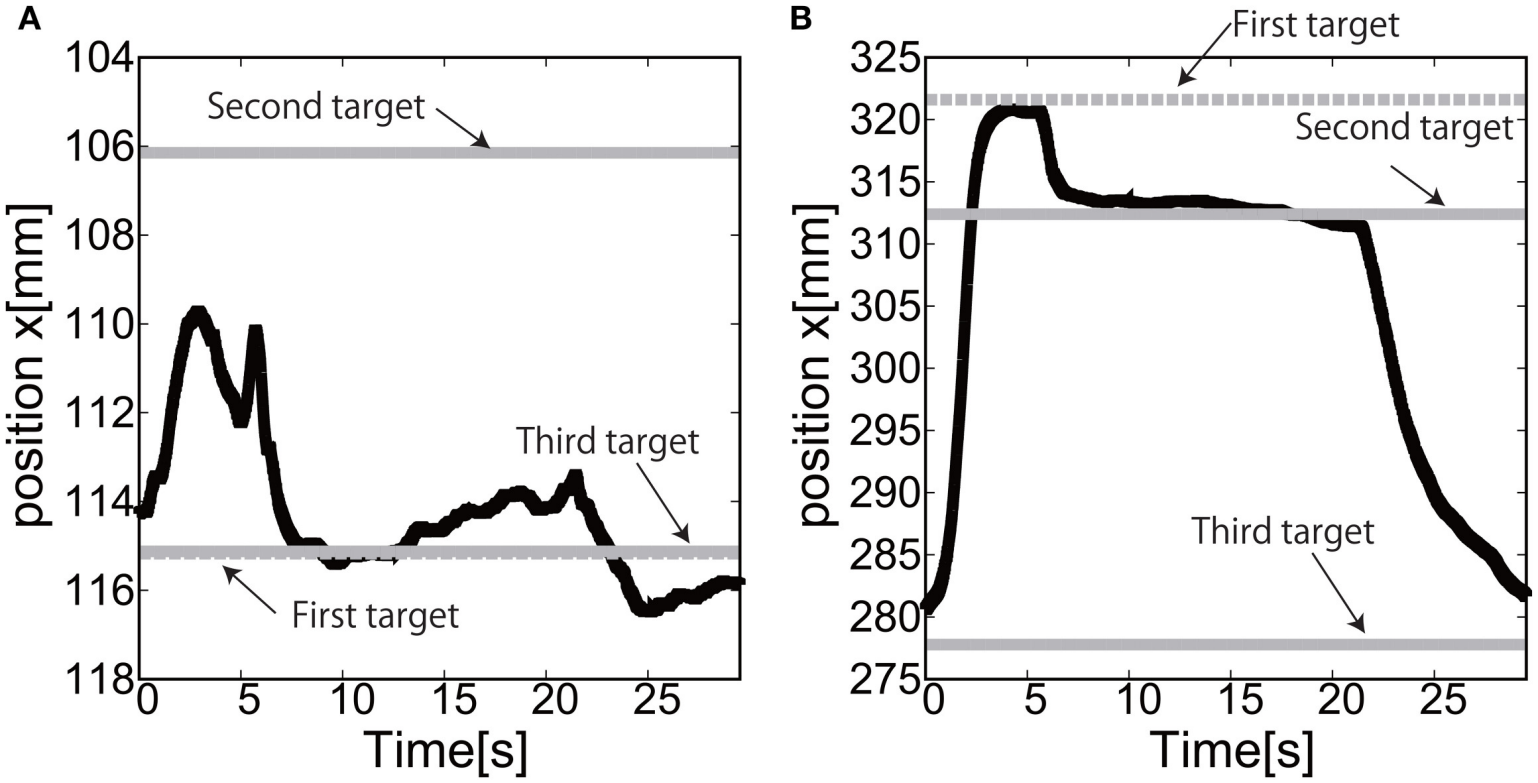
Reaching motion using the elbow and shoulder joint of the Exoskeleton, using the camera on the shoulder. **(A)** *x*(*t*) as a function of time. **(B)** *y*(*t*) as a function of time.

**Figure 25:**
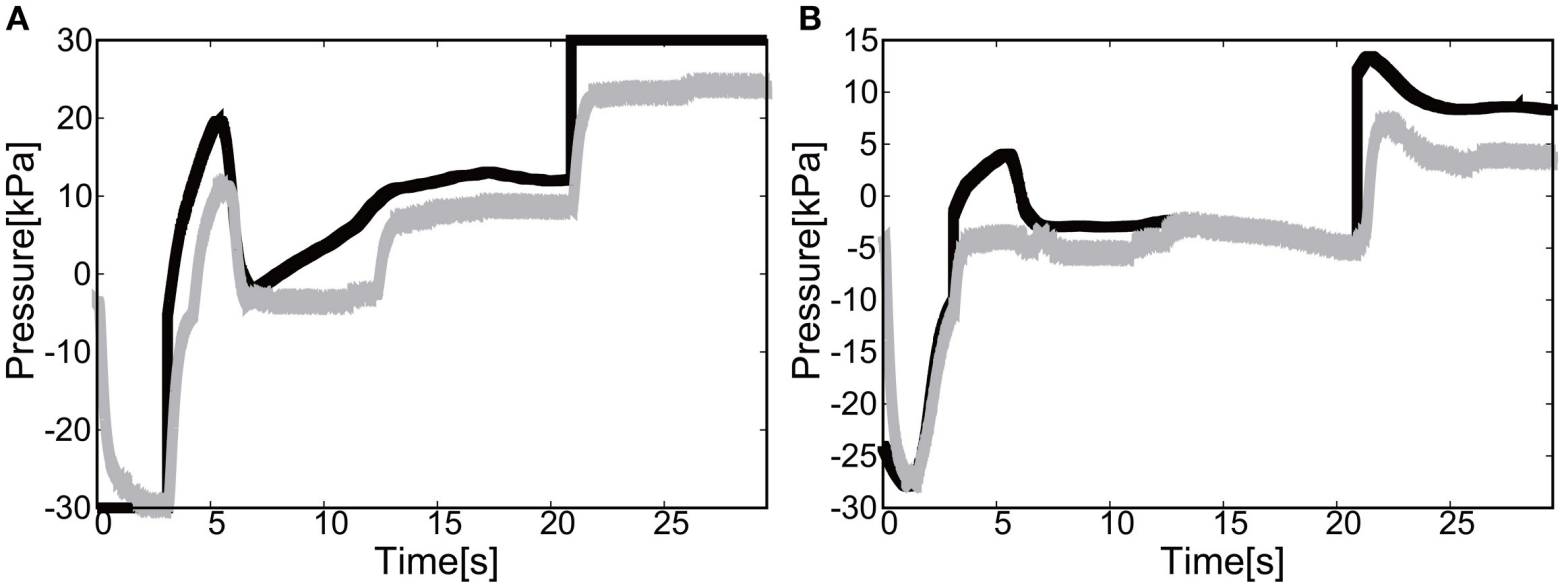
Reaching motion using the elbow and shoulder joint of the Exoskeleton, using the camera on the shoulder. The black line represents the target pressure, and the gray line represents the measured pressure. **(A)** The pressure of the shoulder module as a function of time. **(B)** The pressure of the elbow module as a function of time.

Figures 24A,B show the position of the target, *x*(*t*) and *y*(*t*), using the camera on the shoulder. We can clearly observe that the end-point reaches the first target, the second target, and goes back to the initial position where the third target was located. Figure 25 shows the target and measured pressure as a function of time for the shoulder (a) and the elbow (b) joint. When switching the target position, there were corresponding jumps in the target pressure. Overall, it is a promising feature of the soft module used to actuate the exoskeleton to demonstrate the smooth transition in switching the sign of the pressure, leading to the smooth transition from flexion to extension.

**Figure 26:**
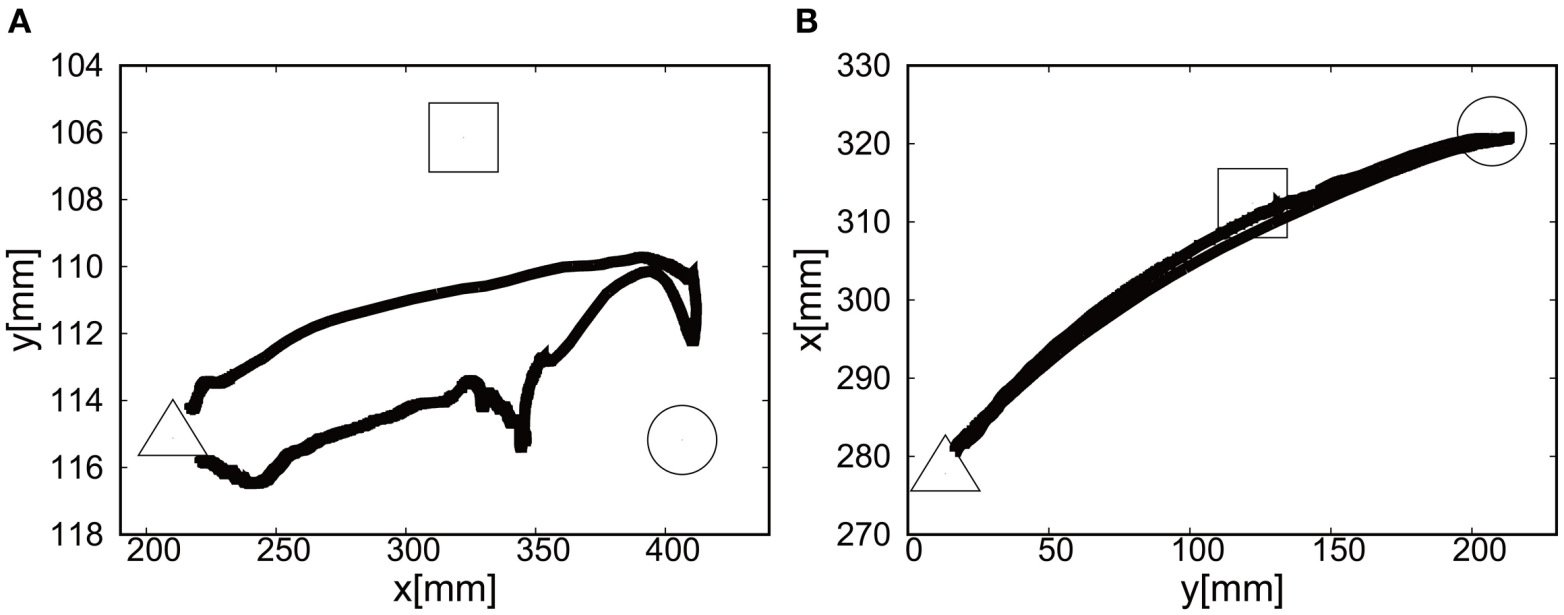
Trajectories of the end-effector in x-y plane, using **(A)** the camera on the shoulder and **(B)** the camera from above. The first target is denoted by circle, the second target by square, and the third target by triangle.

For the case of reaching motion, by switching three targets sequentially it was shown that the EASoftM could demonstrate the closed loop motion as shown in Figure 26, even though there were some overshoots in the relative distance and the measured pressure as a function of time when switching the targets. Note here, that the straight trajectories can be realized by tuning the transformation matrix from visual to work space in Equation (8) (Henry Eberle and Hayashi, 2017).

## 6. General discussion

Therapeutic intervention using the assistive robots has been proven to be effective when including the following design specification (Loureiro et al., 2014);

1. A few degrees of freedom; Motion in daily life requires the coordinated joint motion. Thus, the assistive robots are expected to support a set of joints in upper or lower limbs.
2. Precise motion control: The precision at the reaching point is required, as well as the precision in trajectories and certain dynamical aspects (target velocity and acceleration).
3. Wearable assistive robots in daily life: Cost-effective and ultra-light solutions should be developed for wearable assistive robots which support the motion in daily life.
4. Compliant assistance: Muscle contraction provides compliance in joint dynamics. Thus, assistance should also be compliant.

To realize the precise motion with a few degrees of freedom, the conventional methods would suggest the industrial type robot such as metal exoskeleton which is a hard, rigid structure and heavy (Krebs et al., 2000), not fulfilling the condition 3. A new type of a wearable robot called soft exoskeletons or exosuits is an interesting alternative. Removing the external metal frame, soft exoskeletons become compliant, much lighter, and could be significantly less expensive than rigid frame wearable robots (Panasonic, 2005). However, soft exoskeletons main drawback is the same as their main strength: they have no external rigid frame to support the body parts and transfer force effectively from actuator to some area of body.

We think that the effective transfer of the force should take into account the anatomical structure of limbs and joints, requiring the solid structure of the assistive device. Elderly and disabled people already have weakened bones and muscles, so if the force is transmitted in the wrong manner, adding unnecessary strain to their body parts, it might lead to joint degeneration, damage their muscles or tendons. Thus, in our study, aiming at the reaching motion of upper limb, we developed and validated the exoskeleton actuated by soft modules to fulfill these four conditions. We proposed the exoskeleton aligned with the anatomical structure actuated by the soft modules located at the joint positions, and these soft actuators realized the compliant motion based on pneumatic actuation and visco-elastic properties of soft materials such as plastic and rubber. As far as we are aware of, this proposed structure demonstrated the first possibility of integrating the rigid exoskeleton and the soft actuators to assist the reaching motion, as well as the vision-based control law showing its effective functionality in guiding the hand of the participant to the target.

This vision-based control has a certain advantage when the exoskeleton is used in the daily life, i.e. the visual analysis can be easily extended to detect the target such as cups and pens in the work space, and it does not require any encoders and a set of cables transmitting the signals, which add technical complexity to the integrated system. Thus, closing the control loop only by visual information is a flexible solution that can easily integrate low level motion control with higher level intelligent control. More specifically, since we aim at slow reaching motion, the necessary torque mainly will be used to compensate the gravity. Although, it is in principle possible to use the active actuator for this purpose, however, it would be hard to produce the wearable device aligned with the anatomical structure, as it would increase the number of actuators required. Thus, in our study we used the passive actuator for gravity compensation to decrease the weight for wearable purposes. As a next step, we developed the pneumatic actuators made of plastic sheets, which has good tensile strength and is light, and could produce substantial torque for our purposes under 1 atmospheric pressure. Conventionally, antagonist actuator is necessary to actuate the single joint, resulting in more weight and higher number of tubes. In our study, we could successfully demonstrate that the positive and negative pressure can be applied subsequently to produce the torque to increase and decrease the angle of joints. The low pressure actuation contributed to the smooth transition of the pressure as shown in Figures 6, 7.

Most of the assistive robots were made of rigid links and motors which used metallic materials. To realize the compliant motion, the mechanical structure required additional component such as spring, which also added more weight onto the whole structure (Loureiro et al., 2011). Also, considering the assistive soft robotics, though the pneumatic actuators are light weighted, they were used with the metal links and pulleys, thus, the total weight tend to increase, making them inappropriate for the wearable solution (for example, see Schulz et al., 2011). Our proposed exoskeleton aimed at the ultra-light solution, using the 3D printed links and the soft modules (plastic sheets) for actuation, and reducing the number of actuations by employing the negative pressure actuation and gravity compensation. In addition, despite of the challenges imposed by such choice of hardware components materials, we did not lose the precision of position control since the vision-based control was employed.

The further work is planned to make a whole system portable and wearable. This can be achieved by the pneumatic actuators, the regulators and the power supplies being mounted in a waist belt pack so that the participants can freely move around. Also, aiming at the wearable and portable system, we can use the small motors to pump in the air, and the simple electric circuits to control the pressure of the soft modules (Oguntosin et al., 2015). By placing those electric components in the waist belt, the electromagnetic waves should not interfere with the EEG measurement. In the near future, we will integrate the robotic system with an eye tracking system to transmit the intention of the participant identifying the desired targets, as a natural extension of the vision-based control schemes toward the daily exercise. As a mid-term plan, we will integrate the proposed robotic system with Brain Computer Interface to estimate the user's motion intention in order to trigger the assistive motion (Hayashi et al., 2012; Kondo et al., 2015), as the system discussed in this article provides an optimal solution for the measurement of the EEG signals, since its construction made of plastic (metal-free structure and actuation) in principle does not produce any electromagnetic waves.

## Ethics statement

This study was carried out in accordance with the recommendations of Ritsumeikan medical ethical guidelines and Ritsumeikan Ethical Committee with written informed consent from all subjects (BKC-medical-2016-051). All subjects gave written informed consent in accordance with the Declaration of Helsinki. The protocol was approved by the Ritsumeikan Ethical Committee.

## Author contributions

VO and YM substantially contributed to the development of the systems, data acquisition, and analysis. HK contributed to the performance of experiments, and the interpretation of the results. SN, SK, and YH contributed to the design of the work and overall discussion. All of us contributed to the writing of the paper.

### Conflict of interest statement

The authors declare that the research was conducted in the absence of any commercial or financial relationships that could be construed as a potential conflict of interest.
